# Proteomic consequences of *TDA1* deficiency in *Saccharomyces cerevisiae*: Protein kinase Tda1 is essential for Hxk1 and Hxk2 serine 15 phosphorylation

**DOI:** 10.1038/s41598-022-21414-x

**Published:** 2022-10-27

**Authors:** Henry Müller, Antoine Lesur, Gunnar Dittmar, Marc Gentzel, Karina Kettner

**Affiliations:** 1 Institute of Physiological Chemistry, Technische Universität Dresden, Medizinische Fakultät Carl Gustav Carus, Fetscherstrasse 74, 01307 Dresden, Germany; 2 Luxembourg Institute of Health, 1a Rue Thomas Edison, 1445 Strassen, Luxembourg; 3 Department of Life Sciences and Medicine, University of Luxembourg, 6 Avenue de Swing, 4367 Belvaux, Luxembourg; 4 Center for Molecular and Cellular Bioengineering (CMCB), TP Molecular Analysis / Mass Spectrometry, Technische Universität Dresden, Tatzberg 46/47, 01307 Dresden, Germany

**Keywords:** Kinases, Scientific data, Proteomics, Bioinformatics, Proteomic analysis

## Abstract

Hexokinase 2 (Hxk2) of *Saccharomyces cerevisiae* is a dual function hexokinase, acting as a glycolytic enzyme and being involved in the transcriptional regulation of glucose-repressible genes. Relief from glucose repression is accompanied by phosphorylation of Hxk2 at serine 15, which has been attributed to the protein kinase Tda1. To explore the role of Tda1 beyond Hxk2 phosphorylation, the proteomic consequences of *TDA1* deficiency were investigated by difference gel electrophoresis (2D-DIGE) comparing a wild type and a Δ*tda1* deletion mutant. To additionally address possible consequences of glucose repression/derepression, both were grown at 2% and 0.1% (w/v) glucose. A total of eight protein spots exhibiting a minimum twofold enhanced or reduced fluorescence upon *TDA1* deficiency was detected and identified by mass spectrometry. Among the spot identities are—besides the expected Hxk2—two proteoforms of hexokinase 1 (Hxk1). Targeted proteomics analyses in conjunction with 2D-DIGE demonstrated that *TDA1* is indispensable for Hxk2 and Hxk1 phosphorylation at serine 15. Thirty-six glucose-concentration-dependent protein spots were identified. A simple method to improve spot quantification, approximating spots as rotationally symmetric solids, is presented along with new data on the quantities of Hxk1 and Hxk2 and their serine 15 phosphorylated forms at high and low glucose growth conditions. The Δ*tda1* deletion mutant exhibited no altered growth under high or low glucose conditions or on alternative carbon sources. Also, invertase activity, serving as a reporter for glucose derepression, was not significantly altered. Instead, an involvement of Tda1 in oxidative stress response is suggested.

## Introduction

Eukaryotes have developed eclectic regulatory networks to maintain homeostasis. Glucose metabolism has a central role in these networks as glucose is a key substrate for ATP regeneration and anabolic metabolism as well as the preservation of the intracellular reducing potential^1,2^. Hexokinases not only catalyze the first step of glucose metabolism by hexose phosphorylation but are also involved in glucose sensing and signaling^2,3^.

The *Saccharomyces cerevisiae* (*S. cerevisiae*) genome encodes three known hexose kinases: the two differentially regulated hexokinase isoenzymes Hxk1 and Hxk2, which phosphorylate both aldo- and keto-sugars, and the glucokinase Glk1, which is specific for aldohexoses^4,5^. Hxk2 is the predominant hexose kinase during growth at high glucose conditions^6^. In the presence of high glucose concentrations, a fraction of the cytosolic Hxk2 is translocated to the nucleus, where it participates as part of the Mig1 repressor complex in the repression of multiple genes ("glucose repression"). These include the *SUC2* gene that encodes the repressible invertase^7^ serving as a reporter for glucose repression^8,9^.

The translocation of Hxk2 from the cytosol to the nucleus is regulated by Hxk2 phosphorylation of serine 15^10,11^, which is part of a conserved N-terminal sequence motif (K^7^-M^16^). This motif is essential for the shuttling of Hxk2 between cytosol and nucleus^10^. Under low glucose conditions, nuclear Hxk2 is thought to be phosphorylated at serine 15^11,12^ by Tda1^13,14^ and, in its phosphorylated state, associates with the Xpo1 exportin to be exported into the cytosol^15^. As a result, glucose repression is relieved ("glucose derepression"). When glucose concentrations rise, the cytosolic serine 15 phosphorylated Hxk2 is dephosphorylated by the Glc7/Reg1 phosphoprotein phosphatase complex^10,16^ and imported into the nucleus by the α/β-importin (Kap60/Kap95) pathway^10^.

Like Hxk2, Hxk1 is a phosphoprotein^12^. While deletion of the *HXK2* gene only partly affects glucose repression, it is abolished entirely by the additional absence of *HXK1*^3,17^. Both Hxk1 and Hxk2 show a high degree of similarity with an N-terminal stretch of 24 identical amino acids encompassing serine 15^18^. Therefore, serine 15 of Hxk1 is a likely site for phosphorylation also, and the Hxk2 kinase is a likely candidate for that phosphorylation.

Tda1 has been shown to be the kinase directly phosphorylating Hxk2^13,14^. However, also Snf1 has been reported as direct Hxk2 kinase based on the results of an in vitro assay^10^. While the major goal of the current study was a comprehensive analysis of the role of Tda1 beyond involvement in Hxk2 phosphorylation, it also aimed to either qualify or further back the previous determination of Tda1 as indispensable kinase for Hxk2 phosphorylation in vivo by utilizing independent methods. To this end, a systematic proteomics approach employing two-dimensional difference gel electrophoresis (2D-DIGE) was chosen to compare the proteome of the wild type and the corresponding Δ*tda1* deletion mutant under both high and low glucose conditions. At the same time, the experimental design of the present study allowed to reveal proteomic changes in response to different glucose concentrations.

## Results

### Comparative proteome analysis of the BY4742 wild type and Δtda1 deletion mutant strain

**Figure 1:**
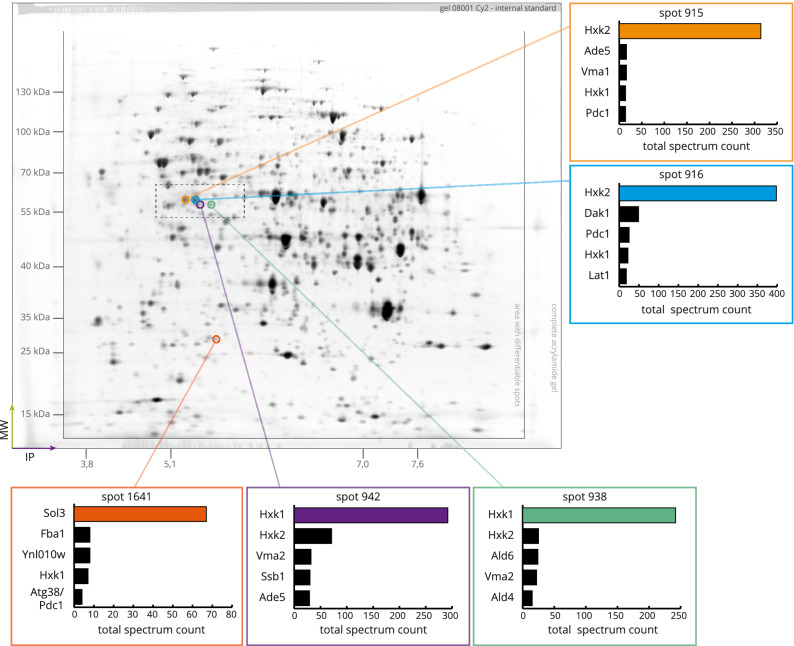
Identification of *TDA1*-dependent proteins by comparison of BY4742 wild type and ∆*tda1* deletion mutant proteomes employing 2D-DIGE. 50 µg of the Cy2 labeled internal standard were separated via 2D-DIGE. The Cy2-channel of the fluorescence scan is shown in the full size of the gel. For each *TDA1*-dependent spot, samples were excised from preparative 2D-gels and, in a blinded experiment, analyzed via LC/ESI–MS/MS. The total spectrum counts from two analyses (separate gels, separate MS runs) were summed for each spot, excluding protein identities only found once. For five spots, the five protein species with the highest spectrum count are depicted in the diagrams. The colored bars in the diagrams indicate the proteins determining the spot identity. The dotted rectangle represents the area that was enlarged for supplementary Fig. S1. Note that the presented gel image was edited for illustrative purposes, and only the area containing the separated proteins is presented with optimized contrast. For the raw image data, see the supplements online at DOI: 10.17605/OSF.IO/7AEQC.

In order to investigate the function of Tda1 and its role in Hxk2 serine 15 phosphorylation, a comparative proteome analysis was conducted by 2D-DIGE, contrasting a wild type and a Δtda1 deletion mutant. Different initial glucose concentrations (0.1% or 2% (w/v), low or high glucose conditions, respectively) in the growth media were used to observe glucose repression or derepression in both the wild type and a Δtda1 deletion mutant. A protein whose intracellular amount changed in response to the *TDA1* gene deletion will subsequently be referred to as a *TDA1*-dependent protein. A glucose-concentration-dependent protein refers to a protein whose intracellular amount changed in response to different initial glucose concentrations (0.1% or 2% (w/v)) in the growth medium. The experimental design and the complete growth regimens are described in the Experimental procedures section. The 2D-DIGE experiments allowed for the separation, detection, and complete matching of 1953 protein spots across a total of 10 gels with three fluorescence color channels each (Melanie software). An example of one such channel from the fluorescence scans of the produced gels can be seen in Fig. 1. Eight spots were identified as *TDA1*-dependent by analysis with the Melanie software and verified by employing the DeCyder software as a second, independent method for data analysis. A spot is referred to as *TDA1*-dependent or glucose-concentration-dependent when the spot volume changed at least by factor 2 depending on the tested condition (fold change ≤ 0.5 or ≥ 2), and that change was significant (q-value ≤ 5%). The spot volume is the integrated fluorescence intensity over a spot area. Significance was determined by ANOVA (for details, see the Experimental procedures section). Interaction effects, where spot volume changes due to the *TDA1* gene deletion differed at high or low glucose conditions, as well as glucose-independent spot volume changes due to the *TDA1* gene deletion were investigated. Spot 942 was the only one to show a significant interaction effect, but it also exhibited glucose-independent spot volume changes due to the *TDA1* gene deletion, as did the other seven *TDA1*-dependent spots.

To identify the proteins contributing to the identified spots, samples of all eight *TDA1*-dependent spots were excised from preparative 2D-gels, analyzed via LC/ESI–MS/MS, and identified as Hxk1, Hxk2, 6-phosphogluconolactonase (Sol3), subunit B of the V-type proton ATPase (Vma2) and DnaJ-like protein 1 (Djp1). The term spot identity is used to refer to the protein that resulted from the mass spectrometric analysis of *TDA1*-dependent or glucose-concentration-dependent spots and most likely represents a *TDA1*-dependent or glucose-concentration-dependent protein. Figure 1 shows the location of the unambiguously identified spots and, for each one, features the five protein species contained within the samples that reached the highest spectrum count. Table 1 summarizes the data on *TDA1*-dependent spots and proteins. The spot identity of the spots 913, 944 and 945 could not unequivocally be narrowed down to one protein. Spots 913 and 944 showed a separate maximum and were identified as individual spots, but they were very closely located to spot 915, which was identified as Hxk2. Spot 945 exhibited high spectrum counts for Vma2 but Djp1 also. Supplementary Fig. S1 shows the position of TDA1-dependent spots with ambiguity regarding spot identity. The full dataset of spot identifications is available in the supplements online at DOI: 10.17605/OSF.IO/7AEQC.

**Table 1:** *TDA1-*dependent proteins of yeast strain BY4742.

Spot number	Spot identity	Fold change 0.1% (w/v) Glucose	Fold change 2% (w/v) Glucose	q-value (Benjamini-Hochberg)	*p*-value (uncorrected)
913	Hxk2, phosphorylated, tail left	0.40	0.52	1.12E−03	4.19E−06
915	Hxk2, phosphorylated, main spot	0.11	0.13	7.40E−08	3.94E−11
916	Hxk2, unphosphorylated, main spot	4.44	3.83	1.67E−05	5.35E−08
938	Hxk1, unphosphorylated, main spot	4.64	3.26	2.91E−06	6.19E−09
942	Hxk1, phosphorylated, main spot	0.14	0.61	2.49E−07	3.13E−10
944	Vma2—V-type proton ATPase subunit B	0.23	0.22	2.96E−06	7.89E−09
945	Vma2—V-type proton ATPase subunit B Djp1—DnaJ-like protein 1	0.42	0.73	5.34E−03	2.28E−05
1641	Sol3—6-phosphogluconolactonase 3	2.45	2.48	2.49E−07	3.97E−10

The listed proteins were determined to be *TDA1*-dependent via 2D-DIGE in the course of the proteomic comparison of BY4742 wild type and Δ*tda1* deletion mutant cells. Spot identification is based on mass-spectrometric peptide analysis (LC/ESI–MS/MS) and subsequent determination of the protein most probable to be causing the change in fluorescence observed via 2D-DIGE. The taken steps are described in detail in the Experimental procedures section. The hexokinases Hxk1 and Hxk2 were identified as spot identities for multiple spots. Therefore, the respective spot identities are supplemented by a short description. The phosphorylation state was hypothesized based on the gel positions and later verified by mass spectrometry. The description as "tail" indicates an individual spot, which appears to be connected to a main spot of higher volume within close proximity. The given fold changes refer to the comparison of protein quantities measured by 2D-DIGE in the Δ*tda1* deletion mutant proteomes and in the wild type proteomes (ratio Δ*tda1*/WT). The q- and *p*-values were calculated by ANOVA for the effect of the *TDA1* deletion, independent of glucose concentration.

The hexokinases Hxk1 and Hxk2 were each found in more than one spot. The different isoelectric points, despite the similar molecular weight of these spots, suggested posttranslational modifications to be present, of which a phosphorylation appeared to be very likely. In the following, the designation of hexokinases as phosphorylated or unphosphorylated refers to the phosphorylation of serine 15 specifically, unless indicated otherwise.

Upon *TDA1* deletion, the volume of spot 915 and thus the quantity of phosphorylated Hxk2 decreased drastically while that of spot 916, unphosphorylated Hxk2, increased. Analogously, the same observation was made regarding Hxk1. Spots with phosphorylated Hxk1 decreased in spot volume, whereas those with unphosphorylated Hxk1 increased. While the amount of Sol3 more than doubled, the spots with Vma2 and Djp1 exhibited a decreased spot volume. A visual comparison contrasting the fold change, p-value and spot volume of the *TDA1*-dependent spots and all the other observed spots is available in the supplementary Fig. S2. The predicted and—based on experimental data—interpolated isoelectric point (IP) and molecular weight (MW) of each spot are available in the supplements online at DOI: 10.17605/OSF.IO/7AEQC.

### Determination of Hxk1 and Hxk2 serine 15 phosphorylation state

**Figure 2:**
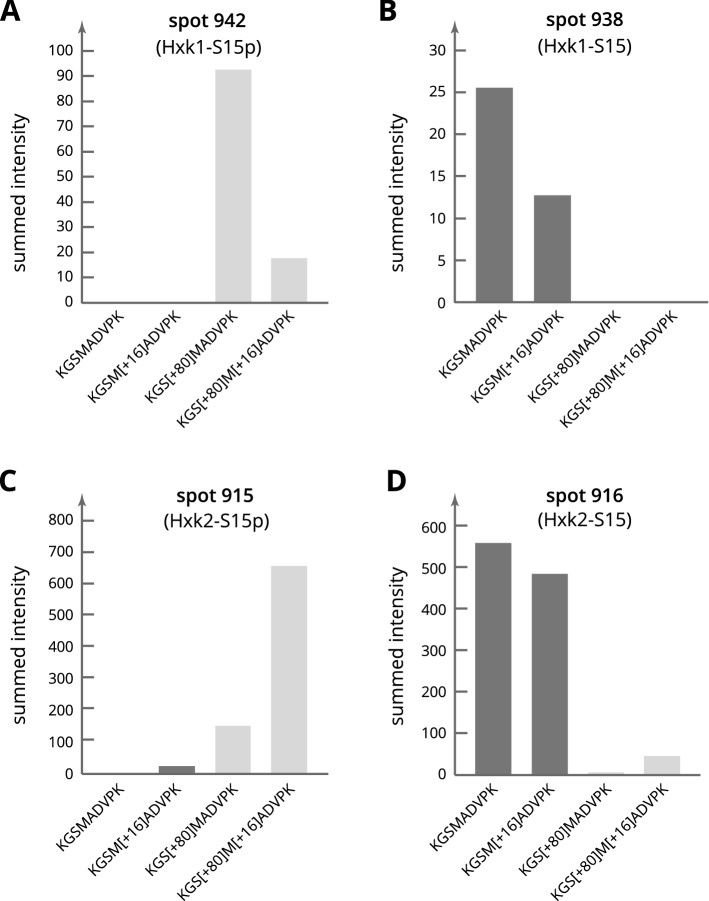
Evaluation of serine 15 phosphorylation in spots with Hxk1 and Hxk2 by PRM. 1 mg of the internal standard, containing aliquots of all proteomes analyzed in the present study, was separated by 2D-gel electrophoresis. For the data shown in (**A**) and (**B**), samples of the Hxk1 main spots (spot 942 and spot 938) were excised from the preparative 2D-gels, digested with trypsin in gel and analyzed by targeted parallel reaction monitoring. For the data shown in (**C**) and (**D**), samples were taken from the Hxk2 main spots (spot 915 and spot 916). The diagrams each show the search for four specific forms of the peptide K^13^GSMADVPK^21^ to evaluate the serine 15 phosphorylation state of the respective protein in each spot.

The divergent IP values of spots 938 and 942 (Hxk1) and spots 915 and 916 (Hxk2) immediately suggested the presence of a posttranslational modification (see Fig. 1 for the spot positions). Particularly in the case of Hxk2, phosphorylation at serine 15, as observed during glucose limitation^11,12^, appeared to be a likely possibility. Considering both the similar patterns of the two pairs of spots essentially consisting of Hxk1 and Hxk2, respectively, and the identical N-terminal 24 amino acids of the two hexokinases, it was postulated that serine 15 phosphorylation may also occur at Hxk1. To test these hypotheses, the N-terminal tryptic hexokinase peptide K^13^GSMADVPK^21^ was specifically examined by targeted proteomics analyses using the parallel reaction monitoring (PRM) method (Fig. 2). Besides searching for phosphorylation of serine (S[+ 80]), the possible oxidation of methionine (M[+ 16]), a common artifact modification found in proteins stored in electrophoresis gels, was taken into account. As expected, spot 916 contained the peptide in its unphosphorylated form almost exclusively, while spot 915 predominantly contained the peptide carrying phosphoserine 15. In spot 942, only the phosphorylated peptide was present. In spot 938, which exhibited only a very low concentration of the peptide, only the unphosphorylated but not the phosphorylated peptide was detected. For MS/MS ion lists, see the supplements online at DOI: 10.17605/OSF.IO/7AEQC. The entirety of PRM data is provided online in the PRoteomics IDEntifications (PRIDE) database with project id: PXD029824.

### Quantification of hexokinase isoenzymes Hxk1 and Hxk2

**Figure 3:**
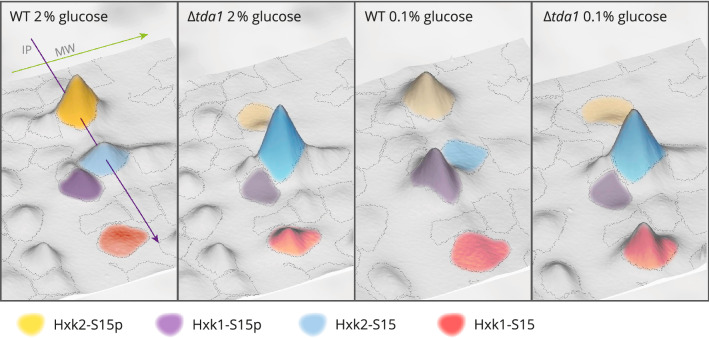
Spots representing Hxk1 and Hxk2 in their phosphorylated and unphosphorylated forms. A proteomic comparison of BY4742 wild type and Δ*tda1* deletion mutant cells grown with different initial glucose concentrations (0.1% or 2% (w/v)) was conducted employing 2D-DIGE. The four main spots containing hexokinases are shown for every combination of conditions. The fluorescence intensities measured by fluorescence scans of the 2D-DIGE gels are presented as elevations from the IP-MW plane. The volume of a spot serves as a measure for quantification of the proteins identified in the spot. Representative images were chosen to provide an intuitive understanding of the spot landscapes, which were also analyzed quantitatively (Fig. 4 and supplementary Fig. S4). The spot borders shown are the ones automatically defined by the Melanie 2D-DIGE analysis software.

The capability to distinguish between the two hexokinases and their phosphorylation state offered, for the first time, the possibility to quantify the different molecular species of the two isoenzymes in a differentiated manner by measuring the volume of the respective spots. Figure 3 provides an intuitive understanding of the 2D-DIGE spot landscape and the volumes which represent the quantities of Hxk1 and Hxk2*.*

After optimizing spot separation and scanning parameters, largely automated fluorescence measurements were utilized as means of quantification first. It was assumed that the fluorescence of the four main hexokinase spots represents the total amount of the two hexokinases in the wild type and the Δ*tda1* deletion mutant under high and low glucose conditions. Surprisingly, as is visualized in supplementary Fig. S3, the total amount of hexokinase appears largely independent of the growth conditions. Assuming normally distributed total amounts, the null hypothesis that the total hexokinase amount of wild type cells grown at an initial glucose concentration of 0.1% or 2% (v/w) would vary by more than 30% can be rejected on a 95% confidence level.

**Figure 4:**
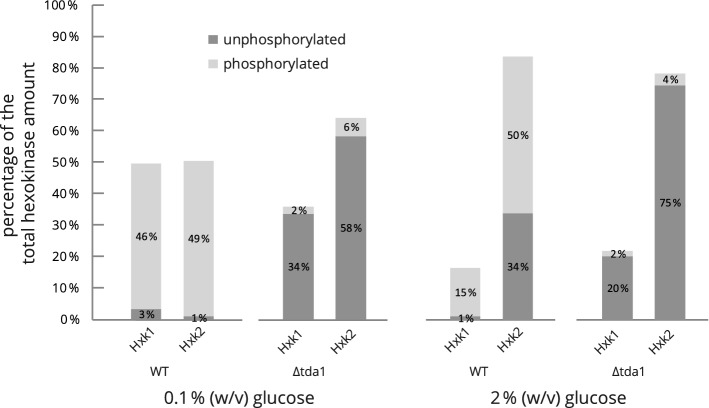
Relative quantification of hexokinase isoenzymes Hxk1 and Hxk2. Based on 2D-DIGE fluorescence data, measurements of the relative amounts of hexokinases present in the wild type and the Δ*tda1* deletion mutant proteomes after cell growth at different initial glucose concentrations (0.1% or 2% (w/v)) were obtained by model-based quantification (for details, see the Experimental procedures section). The spot volumes calculated from the 2D-DIGE fluorescence scans for the main spots containing hexokinases (spots 915, 916, 938, 942) were averaged by calculating the geometric mean and expressed as percentages of the total sum of the four spot volumes. Deviations from the total hexokinase amount of 100% are caused by rounding the displayed values to the nearest integer. See Fig. 3 for an impression of the spot landscapes used as the basis for quantification.

In order to minimize the possibility of inaccurate measurements caused by background fluorescence or poor spot boundary detection, a simple model-based quantification method was established (for details, see the Experimental procedures section). The method was applied for the quantification of hexokinase isoenzymes Hxk1 and Hxk2 in addition to the initial quantification based on the spot volumes obtained by analysis in the Melanie software. Supplementary Fig. S4 shows the relative amounts of both hexokinases based on the analysis in the Melanie software. Figure 8 illustrates the principle of the model-based quantification, where Hxk1 spots on one representative gel were chosen as an example, and, finally, the relative amounts of Hxk1 and Hxk2 that were determined by applying the model-based quantification are presented in Fig. 4.

While the summed volume for all four main hexokinase spots did not vary markedly (supplementary Fig. S3), the distribution of the two hexokinases changed significantly depending on the glucose concentration. In the wild type grown at low glucose conditions, Hxk1 and Hxk2 had about the same quantity; at high glucose conditions, however, Hxk2 dominated with about 84%. Interestingly, in the wild type, the vast majority of Hxk1 was found to be phosphorylated under both high and low glucose conditions. Similarly, unphosphorylated Hxk2 was almost absent at low glucose conditions, but at high glucose conditions, about 40% of the enzyme were unphosphorylated. In the *TDA1* deletion strain, phosphorylation at serine 15 of both hexokinase isoenzymes was lost almost entirely, irrespective of the glucose concentration in the growth medium, showing that *TDA1* is essential for the phosphorylation of both Hxk1 and Hxk2.

### Proteins regulated in response to glucose availability

**Figure 5:**
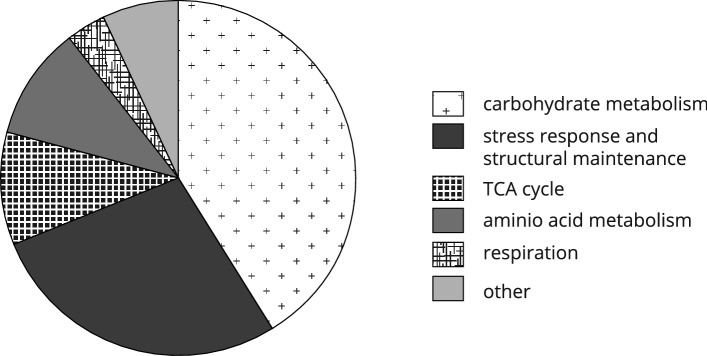
Assignment of glucose-concentration-dependent proteins to functional categories. Glucose-concentration-dependent spots were detected in the fluorescence scans of 2D-DIGE gels by comparing the proteomes of cells grown at different initial glucose concentrations (0.1% or 2% (w/v)) using ANOVA. The glucose-concentration-dependent proteins subsequently identified by LC/ESI–MS/MS were categorized according to their function. Proteins with multiple known functions were assigned to different functional categories accordingly. The category "other" subsumes proteins not considered in any other functional category.

The experimental setup of the 2D-DIGE experiment was chosen with the primary intent to reveal possible interactions of the *TDA1* function and glucose metabolism. Nevertheless, such an interaction was merely detected for spot 942 containing Hxk1-S15p. However, the chosen setup also allowed the detection of several proteomic changes only attributable to the different glucose concentrations. 36 glucose-concentration-dependent spots were found, and 28 different glucose-concentration-dependent proteins were identified via LC/ESI–MS/MS. The complete list of identified spots is provided in supplementary Table S1. The spot positions on the 2D-DIGE gels can be seen in supplementary Fig. S5. The full dataset of spot identifications is available in the supplements online at DOI: 10.17605/OSF.IO/7AEQC.The 28 detected glucose-concentration-dependent protein species were categorized to provide a general overview of the affected cellular functions. The results displayed in Fig. 5 indicate the involvement of about 40% of the detected glucose-concentration-dependent proteins in carbohydrate metabolism, while about one-quarter of the detected proteins is known to fulfill functions in stress response and structural maintenance.

### Growth analyses and measurement of invertase activity

The investigation of the N-terminal protein domain of Hxk2, which contains a nuclear localization sequence^10^ including the serine 15 phosphorylation site, has led to varying results regarding that domain's role in glucose signaling^17,19–23^. This controversy also raises the question of whether the Hxk2 serine 15 phosphorylation is involved in glucose signaling after all. If the regulation of glucose signaling were dependent on hexokinase phosphorylation, the elimination of phosphorylation by *TDA1* deletion could be expected to impair such signaling. One possible indication of impaired signaling and, therefore, possibly less efficient adaption to the environment could be impaired cell growth. However, no significant difference between the growth of the wild type and the Δ*tda1* deletion mutant could be detected under high glucose conditions (supplementary Fig. S6). Under low glucose conditions, only a difference of less than 25 min (*p* ≈ 0.0032), accounting for less than 2% of the overall growth time median (≈ 21.5 h) until reaching an OD_600_ of 1, was observed. Intending to prove equality, one could elaborate that the 95% confidence interval (Wilcoxon matched pairs signed rank test; GraphPad Prism 7) of the growth time until reaching an OD_600_ of 1 revealed deviations from a growth difference of 0 which were as little as below one hour for both high and low glucose conditions. These facts hint that in the yeast strains used and under the conditions tested in the present study, the serine 15 phosphorylation might not be of particular importance for glucose signaling.

After no relevant growth difference on glucose had been observed regarding the *Δtda1* deletion mutant, a closer look was taken at growth on sucrose and invertase activity. Intact glucose repression despite the replacement of serine 15 in Hxk2 by alanine had been reported before (15, 19). In the present study, the absence of Hxk1 and Hxk2 serine 15 phosphorylations was investigated with cells expressing the native hexokinase enzymes, also examining glucose derepression. If phosphorylation of Hxk2 were a prerequisite for disassembly of the Mig1 repressor complex, required for glucose repression, as hypothesized before^24^, one would expect an impaired derepression in Δ*tda1* deletion mutants resulting in reduced growth on sucrose. Interestingly, the growth on sucrose did not reveal any impaired glucose derepression in the Δ*tda1* deletion mutant (supplementary Fig. S7). Invertase activity of wild type and Δ*tda1* deletion mutant cells grown in YPD medium for 4 h was equally repressed at 2% (w/v) glucose and showed the same level of activity at 0.1% (w/v) glucose (supplementary Fig. S8).

**Figure 6:**
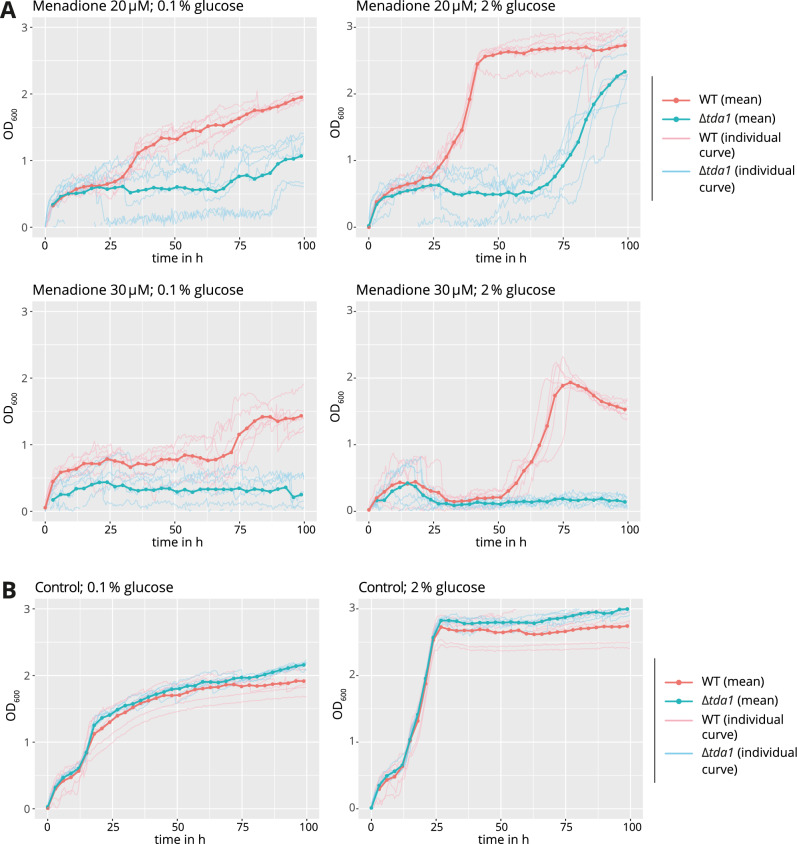
Influence of *TDA1* deletion on resistance against menadione-induced oxidative stress. In (**A**) the growth of the wild type and the Δ*tda1* deletion mutant at different initial glucose concentrations and under oxidative stress by menadione was observed in liquid medium A using a 96-well microplate reader. The control experiments without menadione are shown in (**B**). Per well, 5 μl of cell suspension, OD_600_ 0.08, were added to 200 μl of the medium to test. The data shown are based on two independent experiments, each of which consisted of assays performed in triplicate. Every original curve is shown in addition to the calculated arithmetic means for the time points marked by the dots in the graphs.

In order to explore other possible growth conditions where the physiological function of *TDA1* might be seen phenotypically, growth analyses in 96-well plates were conducted, addressing the question of whether the Δ*tda1* deletion mutant's growth would be impaired under conditions of increased oxidative stress, as had previously been observed for H_2_O_2_ in high throughput experiments^25^. Menadione, known to exert oxidative stress^26^, was tested at various concentrations. In a concentration-dependent manner, menadione led to a prolongation of the lag phase (Fig. 6A,B), whereby the Δ*tda1* mutant was more severely affected than the wild type. These data hint at an involvement of Tda1 in oxidative stress response.

## Discussion

### Proteins regulated in response to the TDA1 deletion

Protein phosphorylation and dephosphorylation represent a universal mechanism of covalent modification that allows cells to modulate structural and functional properties, subcellular localization and complex formation with ligands of their proteins. About 50% of the metabolic proteins in *S. cerevisiae* are reported to be phosphorylated^27–29^, including the hexokinases Hxk1 and Hxk2^12^. In the present exploratory study, the proteomic and functional consequences of *TDA1* deficiency in *S. cerevisiae* were investigated with the aim of also independently verifying the in vivo significance of Tda1 for Hxk2 serine 15 phosphorylation. Phosphorylation of *S. cerevisiae* Hxk2 at serine 15 has four known primary consequences: (i) shift of the monomer-homodimer equilibrium in favor of the monomeric species^30^, (ii) increase of substrate affinity and inhibition by ATP^31^, (iii) higher enzymatic activity^31^, and (iv) translocation of the phosphoenzyme from the nucleus to the cytoplasm^10^. Previous studies revealed that the protein kinase Tda1 is likely responsible for Hxk2 phosphorylation^13,14^.

Proteins from wild type and the respective *Δtda1* deletion mutant grown at high or low glucose conditions to induce glucose repression and derepression, respectively, were subjected to 2D-DIGE. The eight identified *TDA1*-dependent spots were distinguished from all other spots by a strong abundance change and a high significance of the change, while none of them exhibited extreme fluorescence intensities on the edge of the dynamic range (supplementary Fig. S2). By mass spectrometry, the spot identities were determined to be the presumed proteoforms of hexokinase 2 as well as hexokinase 1, the V-type proton ATPase (Vma2), DnaJ-like protein 1 (Djp1) and 6-phosphogluconolactonase (Sol3) (Table 1).

Of the proteins identified beyond Hxk1 and Hxk2, Vma2 represents the non-catalytic subunit of the peripheral V1 complex of vacuolar ATPase, which in a screening of a yeast deletion library was shown to protect against high-O_2_ damage^32^. A function of the V-ATPase in oxidative stress response is also suggested in a study by Charoenbhakdi et al.^33^, according to which it plays a role in the regulation of the cell wall stress response and the prevention of endogenous oxidative stress. Djp1, an abundant yet poorly characterized member of the J-protein/Hsp40 co-chaperone family, is involved in targeting mitochondrial precursor proteins as well as peroxisomal protein import^34,35^. In addition to the remaining questions regarding the detailed function of Vma2 and Djp1, it must be taken into account that spots 944 (Vma2) and 945 (Vma2 and Djp1) are close to neighboring hexokinase spots. Therefore, interpretation needs to be very cautious. The detected abundance changes could partially be attributable to these neighboring spots. Interferences could for example be caused by undetected posttranslational modifications causing a fraction of Hxk1 or Hxk2 to change position on the 2D-gel. The 6-phosphogluconolactonase Sol3 in spot 1641 was unambiguously identified and catalyzes the second step of the pentose phosphate pathway^36^, which, in its oxidative phase, generates D-ribulose 5-phosphate and NADPH. NADPH, among other functions, is needed to combat oxidative stress.

Only one spot with an interaction effect of glucose concentration and genotype was identified. Considering the data shown in Fig. 4, the interaction effect assessed for spot 942 with phosphorylated Hxk1 can be explained as follows: Regarding the wild type, increased glucose availability causes a decrease of phosphorylated Hxk1 as overall Hxk1 is decreased, and most of the enzyme exists in the phosphorylated form. The TDA1 deletion, however, eliminates Hxk1 phosphorylation so that the aforementioned decrease of phosphorylated Hxk1 can no longer be observed. Hence the TDA1 deletion influences the abundance change due to glucose availability.

### Low number of TDA1-dependent proteins

The number of *TDA1*-dependent proteins identified in the present study is remarkably low, possibly reflecting the limits of the chosen 2D-DIGE method. For example, proteins with IP-values below 3 and above 10, such as histones (including histone H3 that has previously been shown to be phosphorylated by Tda1^37^), can not be detected with the chosen setup of the isoelectric focusing. The lack of Tda1 in the Δ*tda1* mutant was also not observable via 2D-DIGE, possibly due to a naturally low number of copies per cell or a split into multiple spots, each below the detection limit, caused by posttranslational modifications. Another reason for the low number of *TDA1*-dependent proteins could be the arbitrary chosen cut-off values for fold change and significance. However, rather arguing against the latter interpretation, the choice of these values seems very appropriate looking at the volcano plot in supplementary Fig. S2. Furthermore, a recent investigation of *TDA1*-dependent proteins by employing a SILAC approach (S. Seiferheld, Technische Universität Dresden, unpublished Data) did not reveal additional proteins with altered abundance. Even though SILAC cannot easily detect the wide range of posttranslational modifications 2D-DIGE can, and highly homologous protein isoforms like Hxk1 and Hxk2 are distinguished with less confidence, the method is typically more sensitive. Thus, it may be that Tda1 serves very particular functions, perhaps of subordinate relevance for survival under the conditions used in the present proteomics study, therefore not causing extensive global cellular changes at the protein level.

### Proteins regulated in response to glucose availability

**Figure 7:**
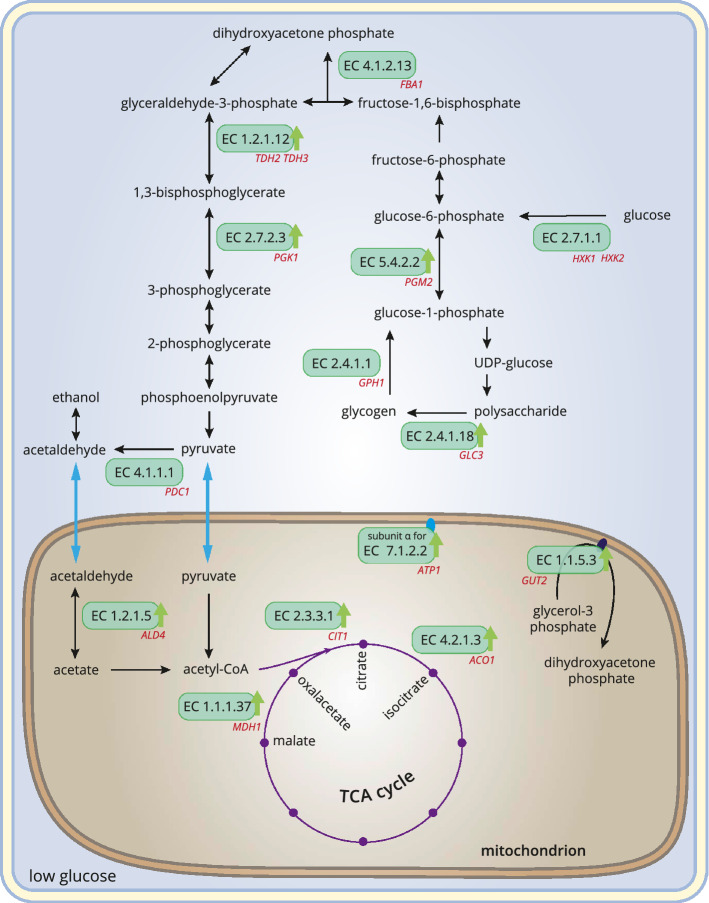
Functional role of glucose-concentration-dependent proteins in carbohydrate metabolism, TCA cycle and respiration. Reactions involving identified glucose-concentration-dependent proteins are represented by their EC numbers. The captions in red name the corresponding genes for the glucose-concentration-dependent proteins identified in the present study. Green arrows indicate likely upregulation at low glucose conditions. Transport processes are indicated by blue arrows, chemical reactions by black ones. For the latter, only a selection of reactants and products is shown.

The experimental setup of the 2D-DIGE allowed the investigation of proteomic changes that can be attributed to different glucose availability in the growth media. 28 proteins varied in their abundance depending on high or low glucose conditions, while most of them were upregulated in response to growth at low glucose conditions (supplementary Table S1). Not surprisingly, many glucose-concentration-dependent proteins fulfill functions in carbohydrate metabolism, and increased quantities of proteins involved in the TCA cycle and respiration were present. As 2D-DIGE cannot provide direct information on the function of the identified proteins, every model for the derived physiological processes must remain speculative and subjective to some extent. Nevertheless in Fig. 7 the attempt was made to provide a graphical overview of where identified glucose-concentration-dependent proteins are involved in carbohydrate metabolism, TCA cycle and respiration.

### Role of TDA1 regarding serine 15 phosphorylation of Hxk1 and Hxk2

While available data provided evidence that Tda1 is responsible for serine 15 phosphorylation of Hxk2^13,14^, this has not been without controversy^10,17,38,39^ as the protein kinase Snf1 had also been reported to fulfill this function^10^ and the nucleo-cytoplasmic translocation of Hxk2 had been found unaltered in the Δ*tda1* deletion mutant^10^. The role of Tda1 in the phosphorylation of Hxk1 has not been investigated before. While the phosphorylation of the peptide K^13^GSMADVPK^21^ in the tryptically digested and phosphopeptide enriched protein extract of yeast cells grown at high glucose conditions had previously been determined to be heavily influenced by the deletion of *TDA1*^40^, the unambiguous assignment of the peptide to either one or both hexokinases had not been possible as the amino acid sequence of the respective tryptic peptide is identical. In the present study, the positions of the *TDA1*-dependent hexokinase spots on the 2D-gels allowed the distinction between Hxk1 and Hxk2 prior to the mass spectrometric analysis of posttranslational modifications. Parallel reaction monitoring of the N-terminal hexokinase peptides (K^13^-K^21^) confirmed serine 15 phosphorylation of both Hxk1 and Hxk2 (Fig. 2). Regarding the Δ*tda1* deletion mutant, the fluorescence signal of spots with the phosphorylated hexokinases was within the margin of background noise (Fig. 3), on the one hand reinforcing that *TDA1* is essential for serine 15 phosphorylation of Hxk2, on the other hand providing evidence that *TDA1* is essential for serine 15 phosphorylation of Hxk1 as well.

### Quantification of hexokinase isoenzymes Hxk1 and Hxk2

The relative quantities of the two hexokinase isoenzymes in their phosphorylated and unphosphorylated form were investigated. Challenges, however, were the interference of neighboring spots on the gel, the extent of background noise in the fluorescence images and imperfections of automatic spot boundary detection. These problems were approached by optimizing spot separation and establishing the model-based quantification method described in the Experimental procedures section. Even though the model-based approach eliminates inaccuracies of common, fully automated quantification software to some extent, arbitrary judgments by the observer bear a risk of introducing bias. However, in the present work, the automated quantification with the Melanie software led to results overall in agreement with the ones obtained by the likely more accurate model-based quantification. The model-based quantification is discussed in more detail in the supplementary comment.

As can be seen in Figs. 3 and 4, the *TDA1* deletion abolished Hxk1 as well as Hxk2 phosphorylation at serine 15, and only minimal relative quantities of the phosphorylated forms were measured, most likely due to remaining spot overlap and background noise. A moderate *TDA1*-dependent decrease of the relative Hxk1 amount was evident at low glucose conditions. Recent data from Oh et al.^37^ identified the histone H3 as a further target of the Tda1 kinase. Tda1 phosphorylates the histone H3 at threonine 11^37^, and threonine 11 phosphorylated histone H3 is enriched at the *HXK1* chromosomal locus upon nutritional scarcity, likely increasing its transcription^37,41^. This may explain the decreased Hxk1 amount upon *TDA1* deletion.

Furthermore, it was shown in the present study that in the wild type grown at high glucose conditions, Hxk2 is the predominant hexokinase, whereas at low glucose conditions Hxk1 and Hxk2 are present at a similar quantity. These results are largely in line with investigations on mRNA levels studied within a timeframe of less than 60 min after the beginning of the glucose limitation, showing a dramatic increase of Hxk1 mRNA while mRNA levels for Hxk2 were drastically decreased^6^. The changes observed on the protein level, in the present study, were relatively moderate compared to the mRNA data. So, one could postulate that the earlier reported short-term response to glucose deprivation^6^ could be followed by a more balanced state, as observed at low glucose conditions in the present study, where the overall Hxk1 and Hxk2 amount is kept near-constant and is comprised of both hexokinases. Levels of glucokinase mRNA have been reported to be upregulated upon glucose deprivation^6^. No evidence of a glucose-concentration-dependent abundance change regarding glucokinase was seen in the present study, which could be attributable to the considerably longer growth time, insufficient detection of the protein via 2D-DIGE or the fact that protein and mRNA levels may not necessarily correlate^42,43^.

Regarding hexokinase phosphorylation, wild type cells grown at high glucose conditions showed only about 60% of Hxk2 to be present in the phosphorylated state, while at low glucose conditions as much as 98% of Hxk2 were phosphorylated. This accords with previous findings^13^. In vitro*,* phosphorylation leads to monomerization of Hxk2 which in turn results in an increase of substrate affinity and higher enzymatic activity^10,31^. Therefore, it would seem plausible if Hxk2 phosphorylation increased the efficiency of glucose utilization under low glucose conditions. However, such a hypothesis does not take into account the overall shift from Hxk2 towards the enzymatically much less active Hxk1^44^ under glucose limitation. It might be possible that, at a constant overall glucose phosphorylation activity as described for *Saccharomyces carlsbergensis*^45^, the observed changes regarding Hxk1 and Hxk2 quantities and phosphorylation promote a different utilization of the generated glucose-6-phosphate. At low glucose conditions, glucose-6-phosphate might be directed less towards glycolysis but more towards other metabolic pathways such as the pentose phosphate pathway, thereby generating more NADPH. Such a mechanism could be beneficial to protect cells from oxidative stress upon increased respiration and would be consistent with a function of Tda1 in oxidative stress response as outlined below. Emphasizing the importance of further research in this regard, the hypothesis of Hxk1 and Hxk2 quantity and phosphorylation influencing the metabolic flux of glucose might offer a teleological explanation why *S. cerevisiae* affords two hexokinases with different phosphorylation, different activity and possibly different interaction with other proteins.

### Role of Tda1 in glucose repression/derepression

Previous data indicate that glucose repression is dependent on the phosphatase Glc7 with its regulator Reg1^46^, and Hxk2 is a target of these two factors^10,16^. Hxk2 or Hxk1 are required for glucose repression^3,17,47^, and a correlation between growth at high glucose availability and decreased hexokinase phosphorylation has been described^12^. As Glc7 reverses Hxk2 serine 15 phosphorylation, one might expect Tda1 as the antagonistic kinase to be involved in glucose derepression. The protein kinase Snf1 is essential for glucose derepression^48^, and recently, Oh et al.^37^ reported that Tda1 activity is upregulated by Snf1-dependent phosphorylation at serine 483 and threonine 484. Hxk2 phosphorylation in Δ*snf1* deletion mutants is significantly reduced^13^**,** and Δ*snf1* deletion mutants exhibit significantly impaired respiratory growth^49^.

To assess possible changes in fitness resulting from the *TDA1* deletion, cell growth was monitored at the same growth conditions used for the proteomic comparisons by 2D-DIGE (supplementary Fig. S6). While *TDA1* deletion abolished phosphorylation of Hxk1 and Hxk2 at serine 15, barely any growth differences were observed. This interesting finding suggests that neither Tda1 nor Hxk1 or Hxk2 serine 15 phosphorylation might be required for glucose derepression. This is also supported by the fact that neither the growth on sucrose (supplementary Fig. S7) nor the invertase activities of the wild type and the Δ*tda1* deletion mutant (supplementary Fig. S8) differed significantly and the proteomic comparisons by 2D-DIGE did hardly yield any interaction effects where the *TDA1* gene deletion would have impacted proteomic consequences of altered glucose availability. In summary, while both Glc7 and Snf1 influence serine 15 phosphorylation of Hxk2, this does not appear to be the mechanism by which glucose repression/derepression is facilitated. A model seems conceivable where low amounts of hexokinases are constitutively present in the nucleus allowing for their participation in glucose repression. The shuttling of greater amounts of Hxk2 to and from the nucleus, which has been linked to the serine 15 phosphorylation state of the enzyme^10^, could instead be the manifestation of a different mechanism with a distinct function also regulated by glucose availability.

### Possible function of Tda1 in resistance to oxidative stress

Growth assays (Fig. 6) in the present study provide evidence that Tda1's function may be related to oxidative stress counteractive mechanisms. At menadione concentrations high enough to impair growth of the wild type, the Δ*tda1* deletion mutant exhibited a retarded transition from the lag to the log phase. The reported proteomic consequences of the *TDA1* deletion are also in line with a role of Tda1 in oxidative stress defense.

Of the *TDA1*-dependent proteins in the present study, both the hexokinases and the 6-phosphogluconolactonase Sol3 enable glucose utilization via the pentose phosphate pathway, the main pathway for regeneration of NADPH^50^ as key reducing equivalent. It has been established before that Hxk2 and its serine 15 phosphorylation are involved in controlling cell cycle, mitochondrial function, oxidative stress resistance and chronological lifespan^51^. In the Phosphopep database^27,40^, the phosphopeptides with the greatest fold change in response to the *TDA1* deletion belong to Gtt3 and Hxk1 or Hxk2. Gtt3, which was not identified in the present study, has been reported to most likely be associated with glutathione metabolism^52^. Sol3, on the other hand, is no known target of Tda1 in the Phosphopep Database^27,40^. The latter finding and the identification of Sol3 in only a single spot on the 2D-gels are not consistent with a role of Sol3 as a Tda1 substrate. Furthermore, the observed 2.5-fold increased Sol3 quantity as a consequence of the *TDA1* deletion is not consistent with a decreased resistance to oxidative stress, since an increased abundance of a pentose phosphate pathway enzyme would be expected to rather enhance oxidative stress resistance by increasing NADPH availability. NADPH serves as a key substrate for several mechanisms combating oxidative stress^53^, and it has been demonstrated that *S. cerevisiae* is capable of increasing NADPH production by redirecting glucose to the pentose phosphate pathway^54^. A mechanism further downstream of hexokinase and histone H3 phosphorylation by Tda1, possibly compensating for defects in oxidative stress response in the *Δtda1* deletion mutant, appears to be the most likely explanation for the observed Sol3 abundance increase.

In summary, an important function of Tda1 might be to integrate signals for an increased need to combat oxidative stress, like scarcity of glucose, and perhaps ensure sufficient availability of NADPH by redirecting the metabolic flux of glucose as a result of hexokinase phosphorylation as well as a shifted balance of Hxk1 and Hxk2. The present study provided new data on the relative quantity of Hxk1 and Hxk2 as well as changes in abundance and phosphorylation of both enzymes depending on growth at high or low glucose conditions. It presents an easily applicable new method for spot quantification, confirming the indispensability of Tda1 for Hxk2 serine 15 phosphorylation and identified Hxk1 as a further probable target of the protein kinase Tda1. A crucial involvement of Tda1 in glucose repression/derepression was shown to be unlikely, while various clues for a function in the realm of oxidative stress response were found. Further research will be necessary to investigate the newly generated hypotheses of the present study, to understand how oxidative stress response is mechanistically connected to Tda1 and hexokinase phosphorylation as one of its main functions, why *S. cerevisiae* truly affords two hexokinases, and why their *TDA1*-dependent phosphorylation differs in response to glucose availability.

## Experimental procedures

### 2D-DIGE experimental design and rationale

In order to provide a systematic comparison of the Δ*tda1* and wild type yeast proteome, the current study employs the method of 2D-DIGE. As a basis for the statistical planning and experimental design, the 2D-DIGE dataset in Ref.^55^, which used an experimental approach similar to the one applied in the present study but investigated *Kluyveromyces lactis (K. lactis)*, was analyzed in order to obtain an estimate of the technical and biological variation which could be expected. Technical variability can be reduced by the 2D-DIGE approach very efficiently and appeared insignificant compared to biological variability. To evaluate proteome changes caused by *TDA1* deletion, both at 0.1% and 2% (w/v) glucose, statistical analysis was chosen to be performed with ANOVA. However, planning was conservatively conducted for pairwise t-tests with FDR correction, which had been used in Ref.^55^. 141 genetical and physical interactions were known for *TDA1* according to the BioGrid database^56^ in January 2018. Compared to 5159 open reading frames for proteins potentially targeted by such interactions (Saccharomyces Genome Database), the former number amounts to 2.73%. It was therefore assumed that 97.27% of proteins could be unaffected in the Δ*tda1* deletion mutant. For sample size estimation with R's ssize.fdr, a standard deviation at the 90th percentile of the planning data (2D-DIGE dataset in Ref.^55^) was chosen, and spot data for the *K. lactis* JA6 wild type strain grown at 0.1% or 2% (w/v) glucose with all spots matched across at least five 2D-DIGE gels were considered. A false discovery rate of 5% and a desired power of 80% were chosen. A factor 2 change in spot volume was regarded as relevant, in the sense of biologically meaningful. By combining the protein extracts of four cell cultures and analyzing 5 pools for each condition (wild type and mutant, each at 0.1% or 2% (w/v) initial glucose concentration) the estimated required sample size of about 20 cell cultures for each comparison was reached. The 20 cultures per condition were sorted by time and divided into 4 groups. One culture out of each of the 4 groups was randomly assigned to one pool. For the four different conditions, cultures were grown in parallel and the same assignments to pools were used for every condition. Pairs of the wild type and Δ*tda1* deletion mutant proteome pools were separated on the same 2D-gel. In supplementary Fig. S9, a graphical overview of the pooling procedure is provided. For inter-gel comparisons an internal standard (for details, see the subsection 2D difference gel electrophoresis) was used as is customary for 2D-DIGE experiments.

### Strains

The S288C related *S. cerevisiae* strains were obtained from Euroscarf in the wild type (WT) BY4742 [MATα, *his*3Δ1, *leu*2Δ0, *lys2*Δ0, *ura3*Δ0] and the Δ*tda1* deletion mutant (Δ*tda1*) BY4742Δ*tda1* [MATα, *his3*Δ1, *leu2*Δ0, *lys2*Δ0, *ura3*Δ0, *tda1*::*KanMX4*] variant. The deletion mutant was verified by sequencing the junction region at the start and end of the *TDA1* knockout cassette.

### Cell culture for 2D-DIGE

Cells of the different strains were grown in medium D consisting of 0.69% (w/v) yeast nitrogen base (YNB) without amino acids supplemented with uracil (20 mg/l) and amino acids (L-arginine 20 mg/l, L-histidine 30 mg/l, L-isoleucine 60 mg/l, L-leucine 60 mg/l, L-lysine 40 mg/l, L-methionine 20 mg/l, L-phenylalanine 60 mg/l, L-threonine 50 mg/l, L-tryptophan 40 mg/l). Liquid cultures were agitated at 200 rpm and 30 °C.

Initially, cells were spread on plates with YPD medium (1% (w/v) yeast extract, 2% (w/v) peptone, 2% (w/v) glucose) containing 2.5% (w/v) agar and were cultivated at 30 °C for 72 h. Liquid cultures followed where the cells were grown in precultures of 5 ml with a starting OD_600_ of 0.01 for 15.5 h at 2% (w/v) glucose (preculture 1) and thereafter 10 ml, start OD_600_ 0.06, for 8 h at 2% (w/v) glucose (preculture 2). The second preculture was subjected to centrifugation (3500 g, 5 min, 30 °C) and washed with medium D supplemented with 0.1% or 2% (w/v) glucose, matching the main culture. The main culture was started at an OD_600_ of 0.001 and grown in 100 ml medium D (500 ml flasks with steel caps). Cells were collected at an OD_600_ between 1 and 1.5 by centrifugation (3500 g, 5 min, 2 °C), washed twice with water, frozen and stored at − 80 °C. To prevent the cells from entering stationary growth, 50% of the starting glucose amount were added from a 20% (w/v) stock solution to the main cultures grown at an initial glucose concentration of 0.1% (w/v) when an OD_600_ of approximately 0.3 and 0.6 was reached, thereby compensating for glucose consumption.

### 2D difference gel electrophoresis

The frozen yeast cells were suspended in 4 ml of 2D-DIGE lysis buffer (Tris/HCl 30 mM, urea 7 M, thiourea 2 M, 4% (w/v) CHAPS, DTT 10 mM, 1 × protease inhibitor mix, pH 9.1) per gram of wet cell mass and disrupted employing a French® Pressure Cell Press (4 runs, 20.000 psi). Further treatment was performed as described in Ref.^55^. An internal standard, generated for matching of 2D-DIGE images and calculation of standardized spot volumes, comprised equal protein amounts of all analyzed proteomes in the present study as well as proteomes of a Δ*snf1* deletion mutant derived from the wild type used (strain BY4742Δ*snf1,* analyzed in a separate study by S. Seiferheld, Technische Universität Dresden). All three strains (WT, Δ*tda1 and* Δ*snf1* deletion mutant) were grown both at high and low glucose conditions in 20 cultures each, so that proteomes of 120 individual cultures contributed to the internal standard. The fluorescence labeling with CyDyes, sample preparation for isoelectric focusing and rehydration of IPG strips were performed as described in Ref.^55^. The cyanine dye Cy2 was used for labeling the internal standard. To avoid labeling bias, Cy3 and Cy5 were each used to label some of the wild type and some of the mutant proteomes. Immobilized pH gradient (IPG) buffer pH 3–10 for nonlinear IPG strips was chosen to match the IPG strips (24 cm, pH 3–10, nonlinear). Isoelectric focusing (IEF) was performed with an Ettan IPGphor 3 unit (GE Healthcare, Munich, Germany) at 20 °C. The following program was executed: linear gradient from 0 to 150 V over 3 h, linear gradient to 300 V over 3 h, linear gradient to 1000 V over 3 h, linear gradient to 10,000 V over 3 h, 10,000 V constant for 95,000 Vh, 500 V constant until further use. The current was limited to 50 μA per IPG strip. Two equilibration steps followed before SDS-PAGE. Each was carried out for 30 min in equilibration buffer (Tris/HCl 50 mM, urea 6 M, 20% (v/v) glycerol, 2% (w/v) SDS, pH 8.8), supplemented with 130 mM DTT for the first step, with 135 mM iodoacetamide for the second one. SDS-PAGE was performed employing gradient gels of 1 × 200 × 255 mm dimension, exponentially ranging from 9 to 13.5% acrylamide. The gels were cast after setting the constant volume in the mixing chamber to 80 ml (initial concentration of 13.5% acrylamide) and prefilling the Ettan DALTsix Gel Caster (GE Healthcare, Munich, Germany) with 140 ml gel solution (13.5% acrylamide). The reservoir chamber contained the gel solution with 8.7% acrylamide. Electrophoresis was executed with an Electrophoresis Power Supply EPS 601 (GE Healthcare, Munich, Germany) at 25 °C with 80 V, 1 W of power per gel and an amperage limit of 10 mA per gel for the first hour and 500 V, 13 W of power per gel and an amperage limit of 40 mA per gel until completion.

### Image acquisition and data analysis

Scanning of the two-dimensional gels was performed utilizing a Typhoon 9410 Variable Mode Imager (GE Healthcare, Munich, Germany) with the image resolution set to 50 μm. The excitation/emission wavelengths were 488 nm/520 nm for Cy2, 532 nm/580 nm for Cy3, and 633 nm/670 nm for Cy5. The acquired images were analyzed by applying both the Melanie 2-D Gel Analysis Software Version 9.1.1 (Genebio, Geneva, Switzerland), referred to as Melanie software, and DeCyder 2-D Differential Analysis Software Version 7.0 (GE Healthcare, Munich, Germany), referred to as DeCyder software, in order to verify the results with a different bioinformatics approach. The analysis performed with the Melanie software required manual gel image alignment, whereas spot detection was fully automated and not manually altered to avoid bias. Normalization was performed utilizing the ratiometric normalization function provided by the Melanie software. Settings for normalization were chosen to perform spot filtering based on intensity, excluding spots with a fluorescence intensity below 50% of the maximum fluorescence intensity and calculating the normalization factor as the median of the volume ratios (volume in the image divided by the volume in the normalization reference image for each spot included in the normalization procedure). The internal standard images were included in the normalization procedure. More details on normalization are available in the software documentation. The statistical analysis based on log-transformed spot volumes was performed in the software R^57^. The analysis in DeCyder was carried out with manual revision using the biological variation analysis module, followed by data export and analysis in R analogous to the analysis of the data exported from the Melanie software. ANOVA was used for testing statistical significance. The experiment was set up with a 2 × 2 factorial design with blocking, two factors differentiating the genotype (WT or Δ*tda1*), two the initial glucose amount for cultivation (0.1% and 2% (w/v) glucose). To adjust *p*-values for multiple comparisons, the method of Benjamini and Hochberg^58^ was applied, setting the false discovery rate to a maximum of 5%. *P*-values corrected for multiple comparisons by the Benjamini–Hochberg procedure^58^ are referred to as q-values. The previously mentioned factor 2 change in spot volume as the criterion for biological relevance of proteomic changes was calculated based on 2D-DIGE fluorescence data employing the measure fold change. The fold change is defined as quotient of the geometric mean of the spot volume of all replicates obtained under condition 1 divided by the geometric mean of the spot volume of all replicates obtained under condition 2. The hypothesis of the *TDA1* deletion influencing the abundance of a protein (independent of the glucose concentration) and the hypothesis of the glucose concentration having an influence on protein abundance (independent of the genotype) were considered separately, hence not additionally correcting for multiple testing inherent to ANOVA. A fold change of ≤ 0.5 or ≥ 2 was used as cutoff for relevant changes of protein quantities. For *TDA1*-dependence, the cutoff had to be reached for either 0.1% or 2% (w/v) glucose; for glucose-concentration-dependence, only the fold change measured for the wild type was considered.

When results obtained by analysis with the Melanie software differed from the analysis with the DeCyder software, the spots were manually reviewed. First, when the q-value was below or equal to the cutoff of 0.05 in only one analysis (DeCyder or Melanie software), the spot was not considered when spot detection was plausible in both analyses or neither one. When it was plausible in only one analysis, the q-value from that analysis was considered. Second, when the strict fold change cutoff of ≤ 0.5 or ≥ 2 was met in DeCyder only, and detection was plausible, the spot was regarded to represent a *TDA1*-dependent or glucose-concentration-dependent protein when the fold change in the Melanie software at least met the 5% loosened cutoffs of approximately ≤ 0.53 or ≥ 1.9. Otherwise, the fold change calculated with the data from the Melanie software took precedence. The classification of *TDA1*-dependent spots was consistent among the Melanie and DeCyder software. Eight of the spots found to be glucose-concentration-dependent based on data from the Melanie software had not been plausibly identified with the DeCyder software. Therefore, these spots were confirmed by manual review. Two spots only fulfilled the criteria for glucose dependence in the analysis conducted with DeCyder but were included due to the marginal difference of fold change to the fold change calculated with the Melanie software, based on which only the loosened cutoffs had been met. Beyond the 36 included glucose-concentration-dependent spots, 14 spots found in the DeCyder software had been classified as false positives due to implausible spot detection in the DeCyder software or not meeting the criteria for glucose-concentration-dependence in the Melanie software, where spot detection was plausible. These spots were disregarded. In this work, the spot numbers assigned by the Melanie software are used exclusively.

### Mass spectrometric identification of proteins in 2D-DIGE spots

Samples of *TDA1-* and glucose-concentration-dependent spots were excised from preparative 2D-gels loaded with 1 mg of internal standard and stained using colloidal Coomassie Brilliant Blue G-250. Prior to excision, spot patterns were visually compared to the internal standard fluorescence images to allow for correct spot localization. Protein identification was then performed via LC/ESI–MS/MS.

In-gel digestion with trypsin and peptide extraction were essentially performed as described previously^59^. Samples were separated by liquid chromatography employing an Ultimate 3000 LC-System, trap column 2 cm × 75 µm Acclaim C18 3 µm and fractionating column 15 cm × 75 µm Acclaim C18 2 µm (all Thermo Fisher Scientific, Waltham, USA). Solvent A (2% acetonitrile, 0.1% formic acid in water) was used for trapping (10 min), a linear gradient (5 min, solvent A + 0–60% solvent B (60% acetonitrile, 0.1% formic acid in water)) for separation. An LTQ-Orbitrap XL ETD (Thermo Fisher Scientific, Waltham, USA), MS R = 60,000, MS/MS = Iontrap (low Resolution) was used for data-dependent acquisition (8 most intensive peptide signals). Charge state deconvolution and deisotoping were not performed. All MS/MS samples were analyzed using Mascot (version 2.6.0, Matrix Science, London, UK), assuming the digestion enzyme trypsin. A custom search database (based on SwissProt 2018/07 database selected for *S. cerevisiae*, 8034 entries) including contaminants and used chemicals was used. Mascot was searched with a fragment ion mass tolerance of 0.60 Da and a parent ion tolerance of 10.0 ppm. Carbamidomethylation of cysteine was specified in Mascot as a fixed modification. Oxidation of methionine and acetylation of the N-terminus were specified as variable modifications. Scaffold (version 4.8.5, Proteome Software Inc., Portland, USA) was used to validate MS/MS-based peptide and protein identifications. Peptide identifications were accepted if they could be established at greater than 95.0% probability by the Scaffold Local FDR algorithm. Protein identifications were accepted if they could be established at greater than 99.0% probability and contained at least 2 identified peptides. Protein probabilities were assigned by the Protein Prophet algorithm^60^. Proteins that contained similar peptides and could not be differentiated based on MS/MS analysis alone were grouped to satisfy the principles of parsimony. Total spectrum counts^61^ were used in order to obtain an approximation of the amount each of the identified proteins constitutes to the overall protein amount in one spot. The total spectrum counts from two analyses (separate gels; separate, blinded mass spectrometry experiments) were summed for each spot, excluding proteins only found in one analysis, and the proteins were sorted according to that sum. A protein was regarded as the spot identity when its spectrum count exceeded twice the count of the next most abundant protein in the spot. Otherwise, the spectrum counts of the most abundant proteins were added until a sum of at least 50% of the total spectrum count of all the proteins present in both analyses of the spot was reached. All proteins contributing to this sum were considered as spot identities. The complete dataset of all identified proteins is provided online at DOI: 10.17605/OSF.IO/7AEQC.

### Determination of the serine 15 phosphorylation state of Hxk1 and Hxk2 by parallel reaction monitoring

Excised spot samples from preparative 2D-gels, loaded with 1 mg of internal standard and stained using colloidal Coomassie Brilliant Blue G-250, were destained in 100 mM ammonium bicarbonate/acetonitrile (1/1, v/v) buffer and further dehydrated by addition of MS-grade acetonitrile (100%). Disulfide bonds were reduced in 10 mM dithiothreitol for 30 min at 55 °C and further alkylated in iodoacetamide 50 mM for 30 min at 25 °C in the dark. Samples were digested overnight with sequencing grade trypsin (Promega, Madison, USA) at 37 °C. After digestion the peptides were extracted from the gel spots by successive incubations in 25 mM ammonium bicarbonate, 25 mM ammonium bicarbonate/ acetonitrile (1/1, v/v), 5% formic acid and 5% formic acid/acetonitrile (1/1, v/v) buffers. Samples were finally vacuum dried and dissolved in 30 µl of 0.05% trifluoroacetic acid and 1% (v/v) acetonitrile in water. The LCMS setup consisted of a Dionex Ultimate 3000 RSLC chromatography system (Thermo Fisher Scientific, Waltham, USA) configured in column switching mode. The mobile phases A and B consisted of 0.1% formic acid in water and 0.1% formic acid in acetonitrile, respectively. The loading phase consisted of 0.05% trifluoroacetic acid and 1% acetonitrile in water. The LC system was operated with a Waters BEH C18 (1.7 µm particles) 100 µm × 10 cm analytical column (Waters, Milford, USA). The loading column consisted of Waters Symmetry C18 5 µm 180 × 20 mm (Waters, Milford, USA). Samples were separated by a gradient ranging from 10% (v/v) B to 40% B in 7 min. Data acquisition was performed by a Q-Exactive HF (Thermo Fisher Scientific, Waltham, USA) operated in parallel reaction monitoring mode (PRM). The peptide K^13^GSMADVPK^21^ and its serine 15 phosphorylated form were targeted as well as the oxidized methionine versions of these peptides. Targeted peptides were selected by the quadrupole with an isolation window of 1 m/z, fragmented in the HCD cell with a normalized collision energy of 25, and the fragments ions were analyzed by the orbitrap at a resolution of 60,000 at 200 m/z. MS data were processed by Skyline software^62^ and the signal of all the relevant b and y fragment ions was extracted, including the neutral losses of the phosphorylation (H_3_PO_4_) and the methanesulfenic acid group (CH_3_SOH) related to the loss of the oxidation from methionine.

### Experimental determination of MW and IP

Pre-stained marker proteins for one-dimensional electrophoresis (PageRuler™ Prestained Protein Ladder, Fermentas, St. Leon-Rot, Germany) were separated on 2D-gels and their molecular weights mapped to their coordinates using the Melanie software. The resulting value pairs were fitted by a third-degree polynomial in R (nls Gauss–Newton algorithm) to allow for the calculation of spot MW values. Determination of the isoelectric point was performed by approximating manufacturer-provided pH measurements characteristic for the used 24 cm nonlinear IPG strips (GE Healthcare, Munich, Germany) with a function that was scaled to the actual spot coordinates to best match a sample of marker proteins for two-dimensional electrophoresis (Sigma-Aldrich, Taufkirchen, Germany). These marker proteins were labeled with Cy3 and Cy5.

### Optimized spot volume measurements by model-based quantification

**Figure 8:**
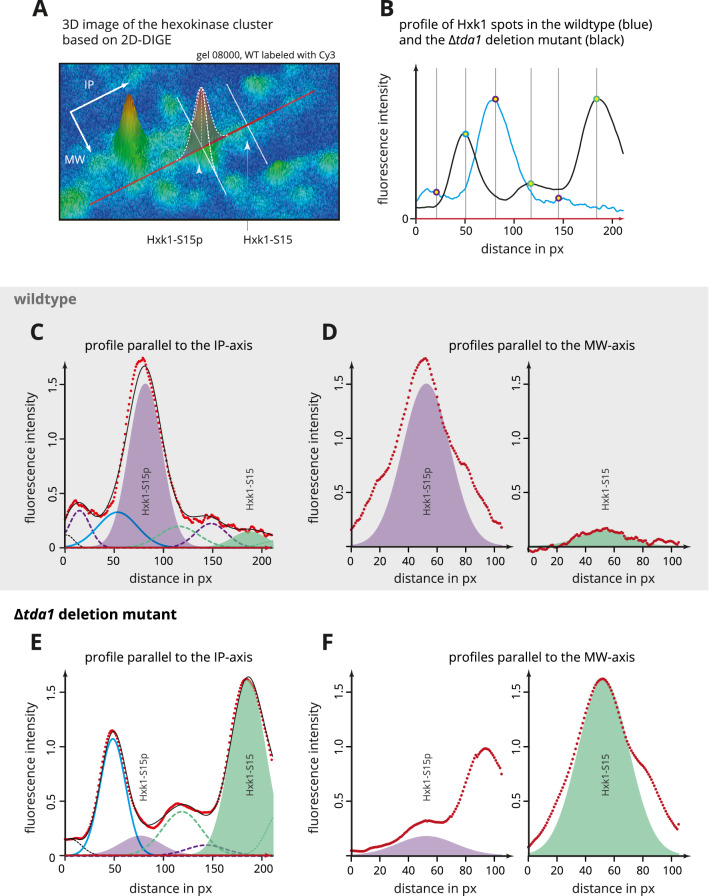
Illustration of the model-based spot quantification. The procedure performed to obtain quantitative information on the relative hexokinase concentrations in the proteome of the yeast *S. cerevisiae* is illustrated exemplarily using two channels of a 2D-DIGE fluorescence scan obtained by separation of fluorescently labeled wild type and Δ*tda1* deletion mutant proteins extracted after cell growth at an initial glucose concentration of 0.1% (w/v). (**A**) visualizes the part of a fluorescence scan of the BY4742 wild type proteome in the region of the hexokinase spots as a 3D landscape. The depicted area approximately corresponds to the area enclosed by the dotted rectangle shown in Fig. 1. Cross-sections of this landscape result in intensity profiles and were obtained with the Fiji image processing package. The red and the two white lines, drawn in parallel to the IP or MW axis, respectively, illustrate the direction of the baseline of these profiles. The profiles were modeled as the sum of Gaussian curves. (**B**) shows the fluorescence intensity profiles of Hxk1 spots along the IP axis for the wild type (blue line) and the Δ*tda1* deletion mutant (black line). The yellow dots mark peaks that were considered to represent individual spots and therefore modeled by one Gaussian curve each. The border line color of the yellow dots matches the color of the corresponding Gaussian curve in (**C**–**F**). The vertical grey lines mark the spot positions chosen as initial values used for the fitting algorithm. The fluorescence data illustrated in (**A**) and (**B**) was modeled as the sum of Gaussian curves. (**C**) and (**E**) show the individual Gaussian curves that resulted from fitting the model to the fluorescence intensity profiles of Hxk1 spots of the wild type and Δ*tda1* deletion mutant proteome. For the Hxk1 main spots, the area under the curve was colored (Hxk1-S15p: purple; Hxk1-S15: green). The spot to which the blue curve belongs, contains Hxk2. The red dots represent fluorescence intensity measurements for each pixel, the overlaid thin black curve the approximation by the fitted model. Shown in (**D**) and (**F**), the calculated Gaussian curves were plotted against the measured fluorescence intensity profiles parallel to the MW axis in order to assess the fit in the second dimension. Again, the red dots correspond to the measurements; the colored area is the area under the Gaussian curve obtained from the model.

In order to measure the changes of the hexokinase quantities on the protein level, beyond the quantification method implemented in the Melanie software, a procedure was applied which is based on modeling the spot landscape in the 2D-fluorescence images by rotationally symmetrical solids with profiles of Gaussian curves (Fig. 8). At the beginning of this procedure, the fluorescence scans were loaded into the image processing package Fiji^63^ (ImageJ version 5.12p) using the Bio-Formats Plugin (Version 5.9.2), and the images were cropped to the area containing protein spots. Prior to exporting the profile data, a 2 px mean filter was applied to smoothen high-frequency noise without changing overall fluorescence. Low-frequency background fluorescence was removed with the sliding parabolic algorithm (35 px), and the average intensity of a spot-free area close to the spots to be analyzed was subtracted. Spot overlap appeared to be greater in the IEF dimension, but peaks were easier to distinguish, so profiles of the spot landscape defined by a line through the maxima of two identified spots approximately parallel to the axis of IEF separation were chosen. The profile data was imported into R and visualized to determine local extrema as shown in Fig. 8B.

The model, which was fitted to the profile data employing the Levenberg–Marquardt algorithm, consisted of summed Gaussian functions. Each function was determined by three parameters to be estimated, one for the height of the curve's peak, one for the position of the center and one controlling the width. The starting and boundary values for the model were calculated identically for all replicates. The entire model was first fit to the fluorescence data of the wild type proteome. Once the center positions of the Gaussian curves had been estimated, these positions were used as initial center positions to fit the model to the fluorescence data of the Δ*tda1* deletion mutant proteome. These center positions were fixed with a tolerance of 500 µm for Hxk1 and 250 µm for Hxk2. From the three parameters of one curve, the volume of the corresponding solid of revolution around the maximum of the curve was calculated. The script implementing the above procedure is available online at DOI: 10.17605/OSF.IO/7AEQC. The resulting volumes for each spot and condition were averaged by calculating the geometric mean and then expressed as percentages of the sum of the four main spots containing hexokinases (spots 915, 916, 938, 942).

### Analysis of the influence of TDA1 deletion and glucose concentration on cell growth

Cell growth was analyzed as the increase of the OD_600_ during the cultivation of cells for the 2D-DIGE experiment. The conditions had been designed to maintain the cells in an exponential growth state until harvesting them for proteome analysis. Therefore, exponential curves could be fitted to the optical densities measured, and the point in time when the culture had an OD_600_ of 1 could be calculated. Statistical testing, comparing the wild type and Δ*tda1* deletion mutant, was performed using Wilcoxon's signed-rank test.

### Analysis of menadione influence on cell growth

The consequences of *TDA1* deletion with respect to the resistance against menadione-induced oxidative stress were studied under different conditions. Cells were grown in medium A (YNB 0.69% (w/v), casamino acids 0.2% (w/v), KOH 150 mg/l, L-tryptophan 20 mg/l, uracil 20 mg/l, carbon source and other additives as indicated) at 30 °C in a FLUOstar OPTIMA microplate reader (BMG LABTECH, Offenburg, Germany) equipped with 96-well BRANDplates® pureGrade (black) and growth was monitored by measuring the OD_600_ every 30 min. Before, cells from YPD plates (YPD medium + 2.5% (w/v) agar) had been grown with medium A in precultures of 5 ml, start OD_600_ 0.15, for 6 h with 2% (w/v) glucose. Subsequently, these cells had been shifted to 11 ml of medium A, start OD_600_ 0.01, and had been grown to their stationary phase. The cells were centrifuged (3500 g, 5 min, 30 °C) and resuspended in medium A without carbon source twice, and the OD_600_ was adjusted to 0.08. Growth was analyzed in 200 μl of the respective medium inoculated with 5 μl of the cell suspension. Menadione was dissolved in 70% (v/v) ethanol. The final amount of ethanol in the analyzed cultures, including controls, was adjusted to 0.3% (v/v).

### Invertase assay

Cells from YPD plates were suspended in 5 ml YPD medium, grown for 16 h to their exponential growth phase, collected by centrifugation (3500 g, 5 min, 30 °C), resuspended in YP medium (1% (w/v) yeast extract, 2% (w/v) peptone) and transferred to 10 ml YP medium supplemented with either 0.1% or 2% (w/v) glucose. Growth was started at an OD_600_ of 0.15 and continued for 4 h. The cultures were agitated at 200 rpm and 30 °C. Invertase activity was assayed essentially as described before^64^.

### Biochemicals and materials

The following substances were used: CyDyes (Cy2, Cy3, Cy5), IPG strips (pH 3–10, nonlinear), IPG buffers (pH 3–10, for nonlinear IPG strips), DeStreak reagent, cover fluid and protease inhibitor mix from GE Healthcare (Munich, Germany); ammonium peroxydisulfate and phosphoric acid from Carl Roth (Karlsruhe, Germany); acrylamide/bisacrylamide solution (37,5:1; 30%), glycine, Tris, and AEBSF from AppliChem (Darmstadt, Germany); DTT from Fermentas (St. Leon-Rot, Germany); urea and RC DC Protein Assay kit from Bio-Rad (Munich, Germany); Coomassie Brilliant Blue G-250 and o-Dianisidine from Serva (Heidelberg, Germany); peptone and yeast extract from BD Biosciences (Franklin Lakes, USA); amino acids, glucose and yeast nitrogen base from ForMedium (Norfolk, United Kingdom); Platinum® Pfx DNA polymerase from Invitrogen (Waltham, USA); Taq DNA Polymerase with Buffers for Enhanced Amplification and Wizard SV Gel and PCR Clean-Up System from Promega (Madison, USA); N,N-dimethylformamide and dipotassium hydrogen orthophosphate from Merck (Darmstadt, Germany); thiourea from Merck (Darmstadt, Germany) or Sigma (Taufkirchen, Germany); PageRuler™ prestained protein ladder from Thermo Fisher Scientific (Waltham, USA); HCl and glycerol from VWR International (Darmstadt, Germany); Peroxidase from Roche Diagnostics (Mannheim, Germany). Other reagents used were purchased from Sigma (Taufkirchen, Germany).

## Supplementary information

This article contains supplementary material in the form of the supplementary Figs. S1–S9, supplementary Table S1 and a comment on the model-based spot quantification citing references ^65,66^.

## Supplementary Information

Supplementary Information.

## Data Availability

The Melanie software project files and all 2D-DIGE fluorescence scans, the predicted and observed IP and MW for each spot, scripts aiding model-based quantification, aggregated data for mass spectrometric spot identification and MS/MS ion lists from targeted proteomics analyses using PRM have been deposited at the Open Science Framework platform, DOI: 10.17605/OSF.IO/7AEQC. Additional primary data for mass spectrometric spot identification has been deposited online in the PRIDE database with project id: PXD037135. The entirety of PRM data has also been deposited online in the PRIDE database with project id: PXD029824. Data on the IPG strip pH gradient, which was used for interpolation of observed IP values, is the property of GE Healthcare and may be provided by them at their sole discretion. Other raw material is available upon request.

## Abbreviations

*S. cerevisiae*: *Saccharomyces cerevisiae*
*K. lactis*: *Kluyveromyces lactis*
2D-DIGE: Two-dimensional difference gel electrophoresis
ANOVA: Analysis of variance
FDR: False discovery rate
Hxk1: Hexokinase isoenzyme 1 of *S. cerevisiae*
Hxk1-S15p: Hexokinase isoenzyme 1 of *S. cerevisiae*, phosphorylated at serine 15
Hxk1-S15: Hexokinase isoenzyme 1 of *S. cerevisiae*, unphosphorylated at serine 15
Hxk2: Hexokinase isoenzyme 2 of *S. cerevisiae*
Hxk2-S15p: Hexokinase isoenzyme 2 of *S. cerevisiae*, phosphorylated at serine 15
Hxk2-S15: Hexokinase isoenzyme 2 of *S. cerevisiae*, unphosphorylated at serine 15
IEF: Isoelectric focusing
IP: Isoelectric point
MW: Molecular weight
OD_600_: Optical density determined at 600 nm/1 cm
YNB: Yeast nitrogen base
