# Supplementary material for: Proteomic consequences of TDA1 deficiency in Saccharomyces cerevisiae: Protein kinase Tda1 is essential for Hxk1 and Hxk2 serine 15 phosphorylation
Source: Sci Rep. 2022 Oct 27;12:18084. doi: 10.1038/s41598-022-21414-x (PMC9613766; doi:10.1038/s41598-022-21414-x)
Supplement: Supplementary file 1 — Supplementary Information. [file 41598_2022_21414_MOESM1_ESM.pdf]

Proteomic consequences of *TDA1* deficiency in *Saccharomyces cerevisiae*

Supplementary Material

Supplementary Tables

Table S1 – Glucose-concentration-dependent proteins of yeast strain BY4742

The listed proteins were determined to be glucose-concentration-dependent via 2D-DIGE in the course of the proteomic comparison of cells grown at an initial glucose concentration of 0.1 % or 2 % (w/v). Significance was determined by ANOVA for the independent effect of the glucose concentration. Spot identification is based on mass-spectrometric peptide analysis (LC/ESI-MS/MS) and subsequent determination of the protein most probable to be causing the change in fluorescence observed via 2D-DIGE. The taken steps are described in detail in the Experimental procedures section. For some spots with ambiguity regarding spot identity, multiple entries exist. The column for functional assignment of the identified proteins uses the following abbreviations: carbo – carbohydrate metabolism; stress – stress response and structural maintenance; tca – TCA cycle; amin – amino acid metabolism; resp – respiration. The given fold changes refer to the comparison of protein quantities measured by 2D-DIGE in the proteomes of the wild type grown at 0.1 % glucose and the wild type grown at 2 % glucose (ratio 0.1 % / 2 % (w/v) glucose). The percentage of spectra attributable to the listed protein is given relative to the total spectrum count of proteins identified in both of two independent LC/ESI-MS/MS experiments conducted for spot identification.

spot number	spot identity	Uniprot ID	functional category	fold change	percen-tage of spectra
389	Gph1 – Glycogen phosphorylase	P06738	carbo	2.05	30.2
389	Hsp104 – Heat shock protein 104	P31539	stress		25.9
390	Fas2 – Fatty acid synthase subunit alpha	P19097	other	2.37	25.3
390	Gph1 – Glycogen phosphorylase	P06738	carbo		25.3
494	Eft1 – Elongation factor 2	P32324	other	2.59	73.2
504	Eft1 – Elongation factor 2	P32324	other	2.31	74,4

568	Met6 – 5-methyltetrahydropteroyltriglutamate-homocysteine methyltransferase	P05694	amin	2.32	57.2
585	Eft1 – Elongation factor 2	P32324	other	2.12	74.8
640	Aco1 – Aconitate hydratase, mitochondrial	P19414	tca	2.59	80.3
671	Glc3 – 1,4-alpha-glucan-branching enzyme	P32775	carbo	2.52	100.0
676	Aco1 – Aconitate hydratase, mitochondrial	P19414	tca	2.32	60.3
774	Pgm2 – Phosphoglucomutase 2	P37012	carbo	1.99	66.4
786	Gut2 – Glycerol-3-phosphate dehydrogenase, mitochondrial	P32191	carbo	2.60	69.6
788	Pgm2 – Phosphoglucomutase 2	P37012	carbo	3.28	43.1
919	Ald4 – Potassium-activated aldehyde dehydrogenase, mitochondrial	P46367	stress	5.72	62.4
938	Hxk1 – Hexokinase 1	P04806	carbo	3.06	63.2
942	Hxk1 – Hexokinase 1	P04806	carbo	7.35	49.2
945	Vma2 – V-type proton ATPase subunit B	P16140	stress	2.51	29.4
945	Djp1 – DnaJ-like protein 1	P40564	stress		22.4
947	Vma2 – V-type proton ATPase subunit B	P16140	stress		23.2
947	Hxk2 – Hexokinase 2	P04807	carbo	2.50	22.6
947	Hxk1 – Hexokinase 1	P04806	carbo		17.2
974	Ssa1 – Heat shock protein SSA1	P10591	stress	2.99	31.1
974	Ssa2 – Heat shock protein SSA2	P10592	stress		30.9
978	Cys4 – Cystathionine beta-synthase	P32582	amin	1.91	28.3
978	Eft1 – Elongation factor 2	P32324	other		27.9
1013	Atp1 – ATP synthase subunit alpha, mitochondrial	P07251	resp	2.06	94.5

1015	Atp1 – ATP synthase subunit alpha, mitochondrial	P07251	resp	2.88	41.7
1072	Pdc1 – Pyruvate decarboxylase isozyme 1	P06169	carbo<br>amin	2.23	85.6
1111	Cit1 – Citrate synthase, mitochondrial	P00890	tca	2.35	35.7
1158	Pdc1 – Pyruvate decarboxylase isozyme 1	P06169	carbo<br>amin	2.07	46.5
1337	Pgk1 – Phosphoglycerate kinase	P00560	carbo	2.42	39.3
1388	Pgk1 – Phosphoglycerate kinase	P00560	carbo	2.52	53.6
1493	Tdh2 – Glyceraldehyde-3-phosphate dehydrogenase 2	P00358	carbo	0.49	48.0
1493	Tdh3 – Glyceraldehyde-3-phosphate dehydrogenase 3	P00359	carbo		36.3
1523	Tdh3 – Glyceraldehyde-3-phosphate dehydrogenase 3	P00359	carbo	2.49	39.5
1523	Tdh2 – Glyceraldehyde-3-phosphate dehydrogenase 2	P00358	carbo		29.8
1533	Mdh1 – Malate dehydrogenase, mitochondrial	P17505	tca	2.96	40.5
1629	Tfs1 – Carboxypeptidase Y inhibitor	P14306	stress	2.33	42.3
1778	Tdh3 – Glyceraldehyde-3-phosphate dehydrogenase 3	P00359	carbo	2.52	91.4
1849	Tdh3 – Glyceraldehyde-3-phosphate dehydrogenase 3	P00359	carbo	2.28	73.3
1889	Tdh3 – Glyceraldehyde-3-phosphate dehydrogenase 3	P00359	carbo	2.08	69.4
1906	Fba1 – Fructose-bisphosphate aldolase	P14540	carbo	2.54	45.3
1924	Tdh3 – Glyceraldehyde-3-phosphate dehydrogenase 3	P00359	carbo	2.45	37.7
1939	Hsp12 – 12 kDa heat shock protein	P22943	stress	4.33	23.7

Supplementary Figures

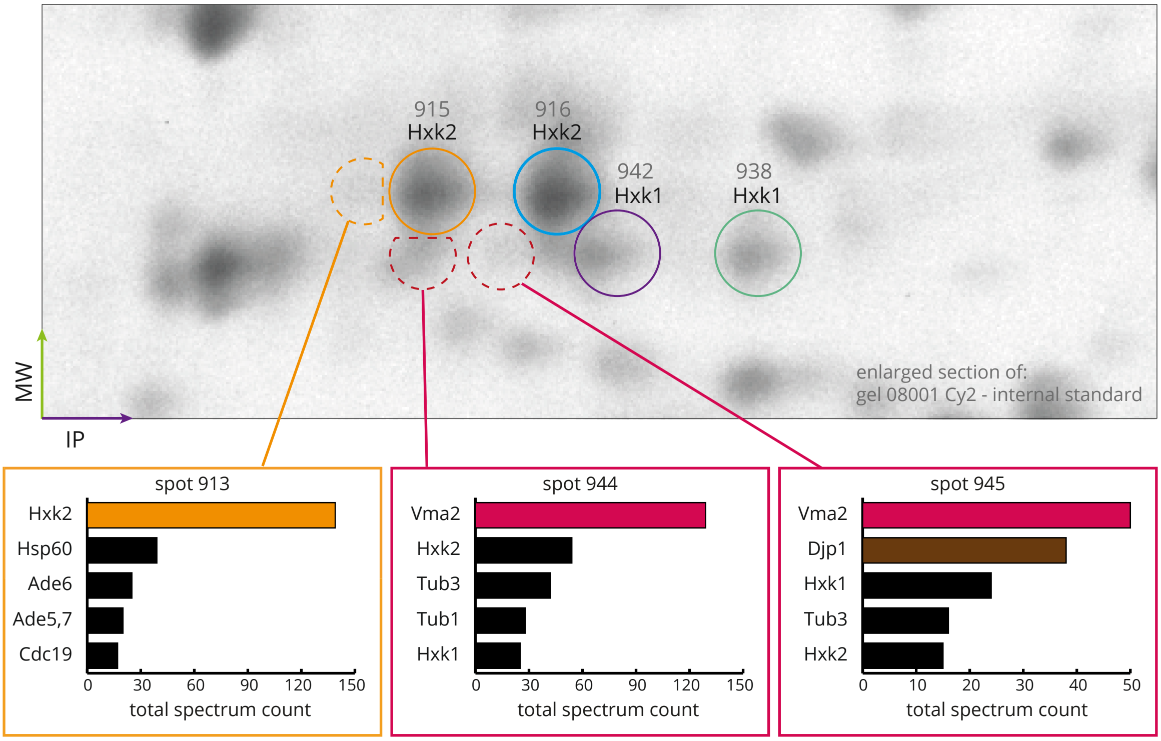

Figure S1 – *TDA1*-dependent spots with ambiguity regarding spot identity resulting from the comparison of the BY4742 wild type and Δ*tda1* deletion mutant proteomes employing 2D-DIGE

The figure shows the enlarged segment of the 2D-DIGE image presented in Fig. 1 where most changes due to the *TDA1* deletion have been detected. 50 µg of the Cy2 labeled internal standard were separated via 2D-DIGE. For each *TDA1*-dependent spot, samples were excised from preparative 2D-gels and, in a blinded experiment, analyzed via LC/ESI-MS/MS. The total spectrum counts from two analyses (separate gels, separate MS runs) were summed for each spot, excluding protein identities only found once. The colored bars in the diagrams indicate the proteins determining the spot identity. The spot identity of the shown spots 913, 944 and 945 could not unequivocally be narrowed down to one protein: Spots 913 and 944 show a separate fluorescence maximum and were identified as individual spots, but they are very closely located to spot 915, which was identified as Hxk2. Spot 945 exhibited high spectrum counts for Vma2 but Djp1 also. Note that the presented gel image was edited for illustrative purposes. For the raw image data, see the supplements online at DOI: 10.17605/OSF.IO/7AEQC.

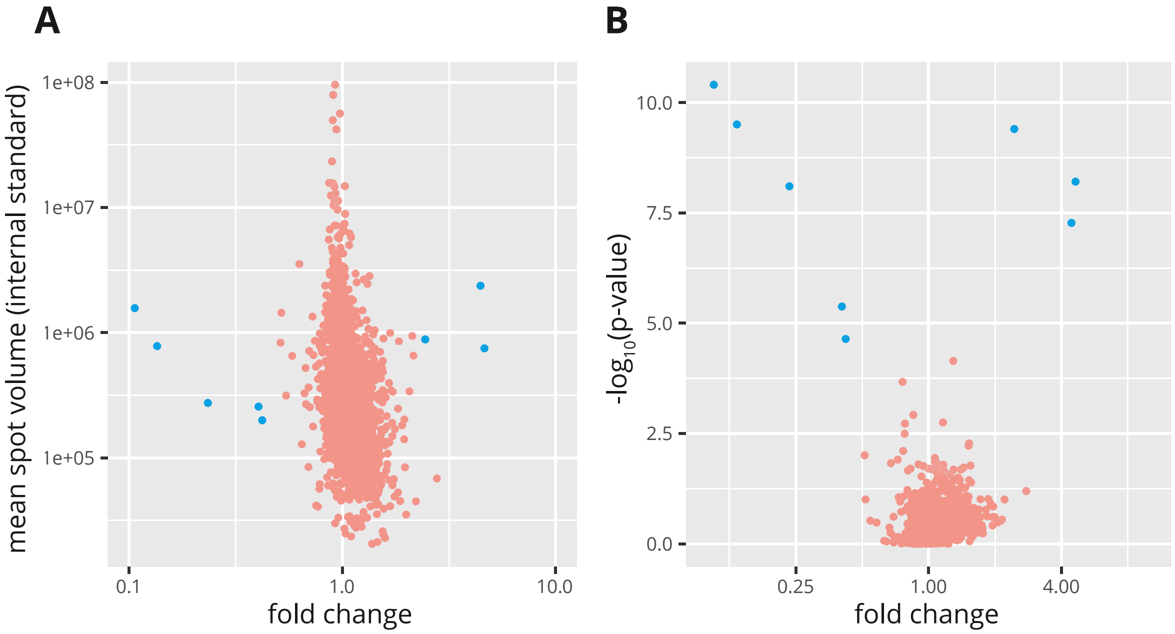

Figure S2 – Visual assessment of possible bias caused by spot volume (A) and volcano plot for assessment of the chosen cutoff values for significance and relevance of abundance changes upon *TDA1* deletion (B)

Differentially labeled proteomes of the wild type and the Δ*tda1* deletion mutant were separated via 2D-DIGE together with an internal standard. Fluorescence scans with the three channels, one for each of the labeling dyes, were loaded into the Melanie software for spot detection and normalization. The resulting normalized intensities in the form of spot volumes were further processed in R. *TDA1*-dependent spots are represented by blue dots. The plots are only shown for the growth at low glucose conditions. Plots obtained with the same procedure for cells grown at high glucose conditions (not shown) were similar. (**A**) visualizes the dynamic range of spot volumes determined by 2D-DIGE and shows that the *TDA1*-dependent spots did not have a volume (calculated as the geometric mean of the fluorescence intensities measured for the internal standard) that was either extremely low or high, which would raise suspicion for bias. (**B**) visualizes the fold change and p-value for every spot. Spots represented by the blue dots appear visually separated from the mass of spots regarded as irrelevant, implying that the cutoff values for significance and relevance defined before the study do not, in combination with the chosen study design, lead to artificially excluded or included spots.

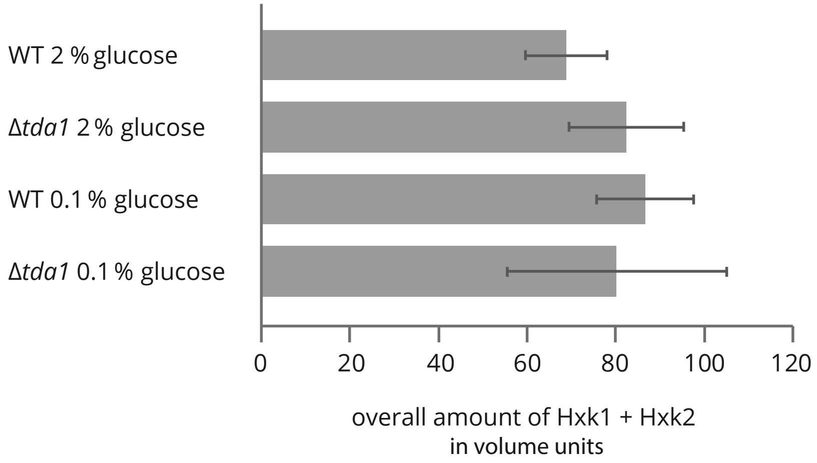

Figure S3 – Relative quantification of overall hexokinase abundance

For the main spots containing hexokinase (spots 915, 916, 938, 942), the normalized spot volumes were exported out of the Melanie software and summed before calculating the arithmetic mean, which is represented by the grey bars. Results are based on 20 cell cultures and five 2D-DIGE fluorescence scans for each combination of conditions represented by one grey bar. The error bars show the standard deviation.

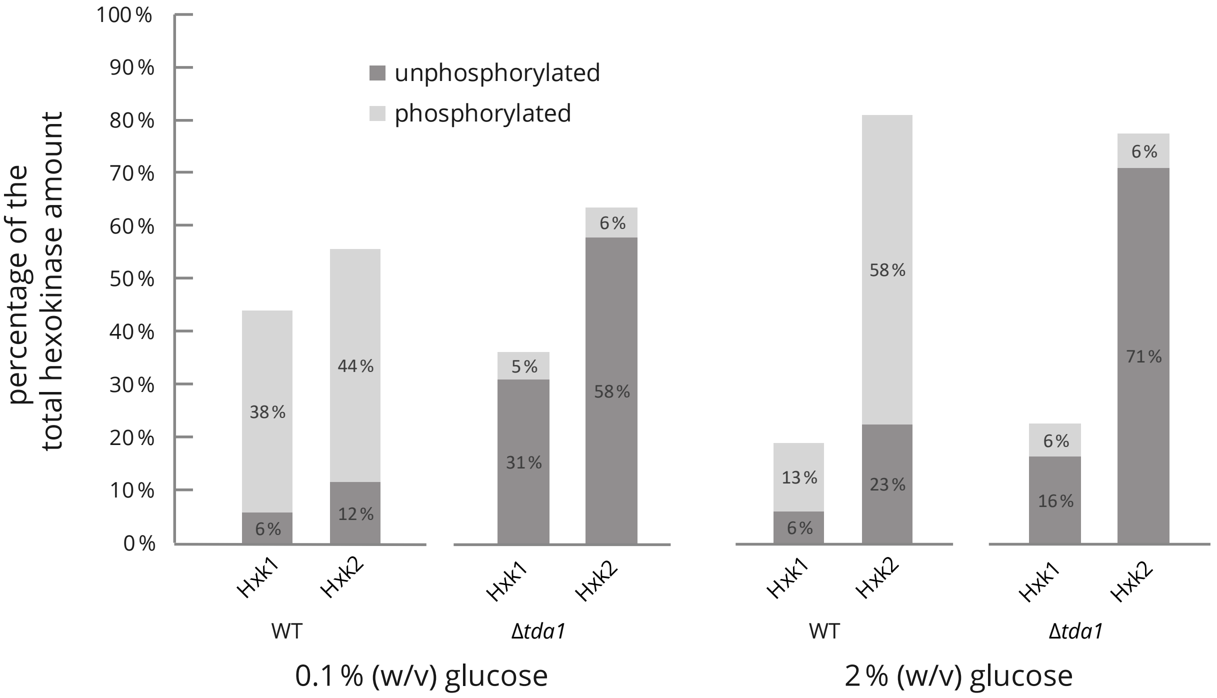

Figure S4 – Relative quantification of Hxk1 and Hxk2 in their phosphorylated and unphosphorylated forms based on quantification with the Melanie software

Based on 2D-DIGE fluorescence data, measurements of the relative amount of hexokinases present in the wild type and the Δ*tda1* deletion mutant proteomes after cell growth at different initial glucose concentrations (0.1 % or 2 % (w/v)) were obtained by analysis in the Melanie software. The spot volumes calculated from the 2D-DIGE fluorescence scans for the main spots containing hexokinases (spots 915, 916, 938, 942) were averaged by calculating the geometric mean and expressed as percentages of the total sum of spot volumes of the four hexokinase species. Deviations from the total hexokinase amount of 100 % are caused by rounding the displayed values to the nearest integer. See Fig. 3 for an impression of the spot landscapes used as the basis for quantification.

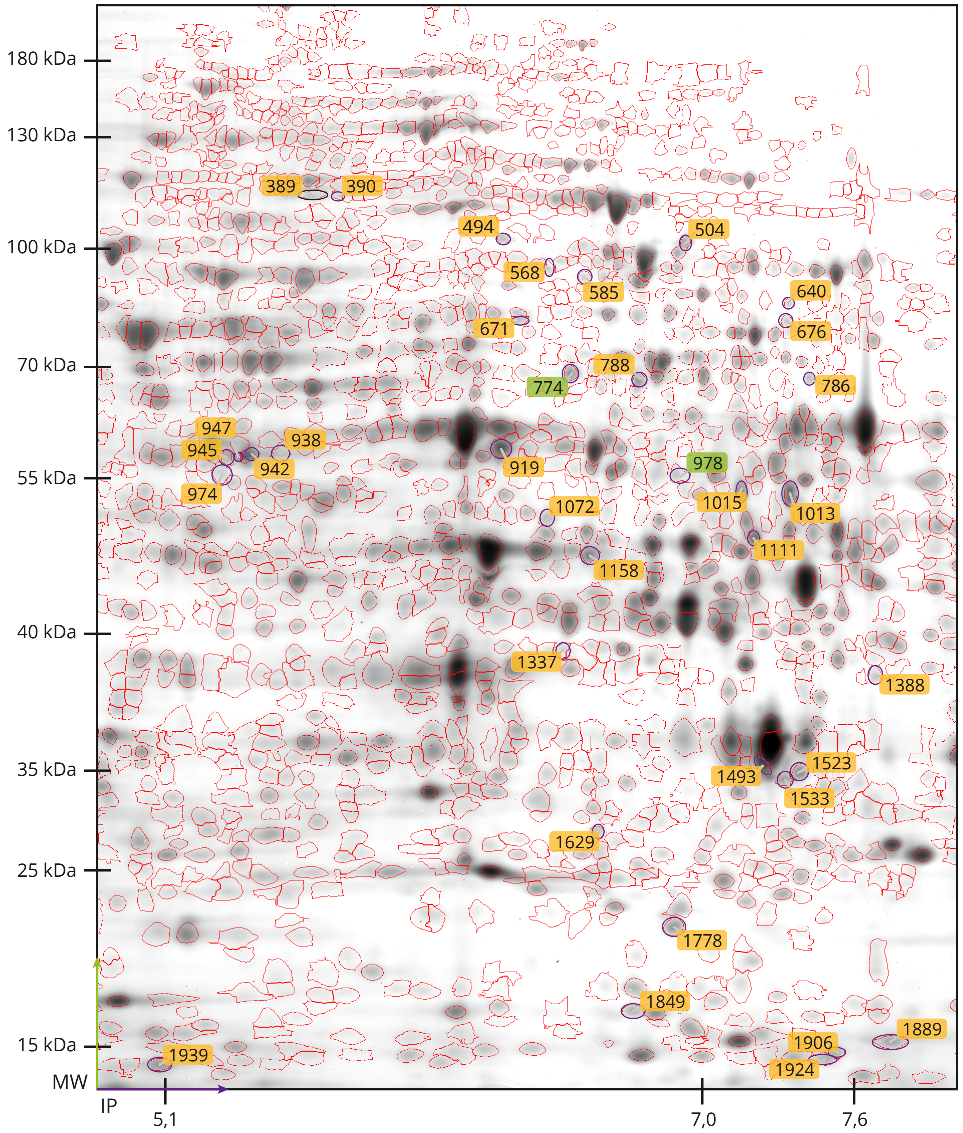

Figure S5 – Glucose-concentration-dependent spots marked in a section of the image of the 2D-DIGE separated internal standard

50 µg of the Cy2 labeled internal standard were separated via 2D-DIGE. A section of the full picture is shown where all spots determined to be glucose-concentration-dependent are labeled. Spots 774 and 978 only meet the strict criteria for glucose dependence in the analysis conducted with the DeCyder software (for details, see the Experimental procedures section). The spot identities for each spot are listed in Table 2. Note that the presented gel image was edited for illustrative purposes. For the raw image data, see the supplements online at DOI: 10.17605/OSF.IO/7AEQC.

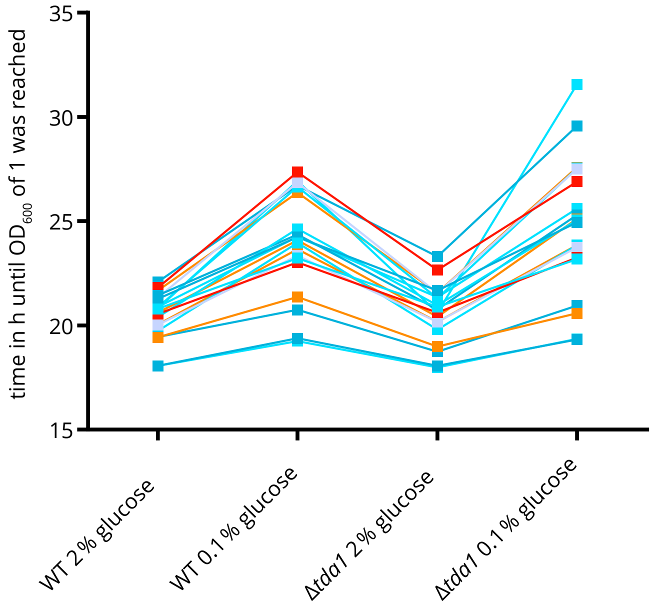

Figure S6 – Growth time until an OD_{600} of 1 was reached in the Δ*tda1* deletion mutant and the wild type

The cell growth of the wild type and the Δ*tda1* deletion mutant were analyzed after growth at initial glucose concentrations of 0.1 % and 2 % (w/v) by measuring the increase in optical density during the cultivation of cells for the 2D-DIGE experiment. Exponential curves were fitted to the measurements to interpolate the time when the culture had an OD_{600} of 1. Each dot represents the time after which one particular culture had reached the OD_{600} of 1. Four cultures grown in parallel are shown connected by a line. Statistical testing comparing the Δ*tda1* deletion mutant and the wild type was performed using Wilcoxon's signed-rank test. At high glucose conditions, there was no significant difference. At low glucose conditions, a significant ($p \approx 0.0032$) difference of 0.41 h median growth time was detected. The 95 % confidence interval (Wilcoxon matched-pairs signed-rank test; GraphPad Prism 7) indicated deviations of growth difference from 0, which were as little as below one hour for an initial glucose concentration of both 0.1 % and 2 % (w/v).

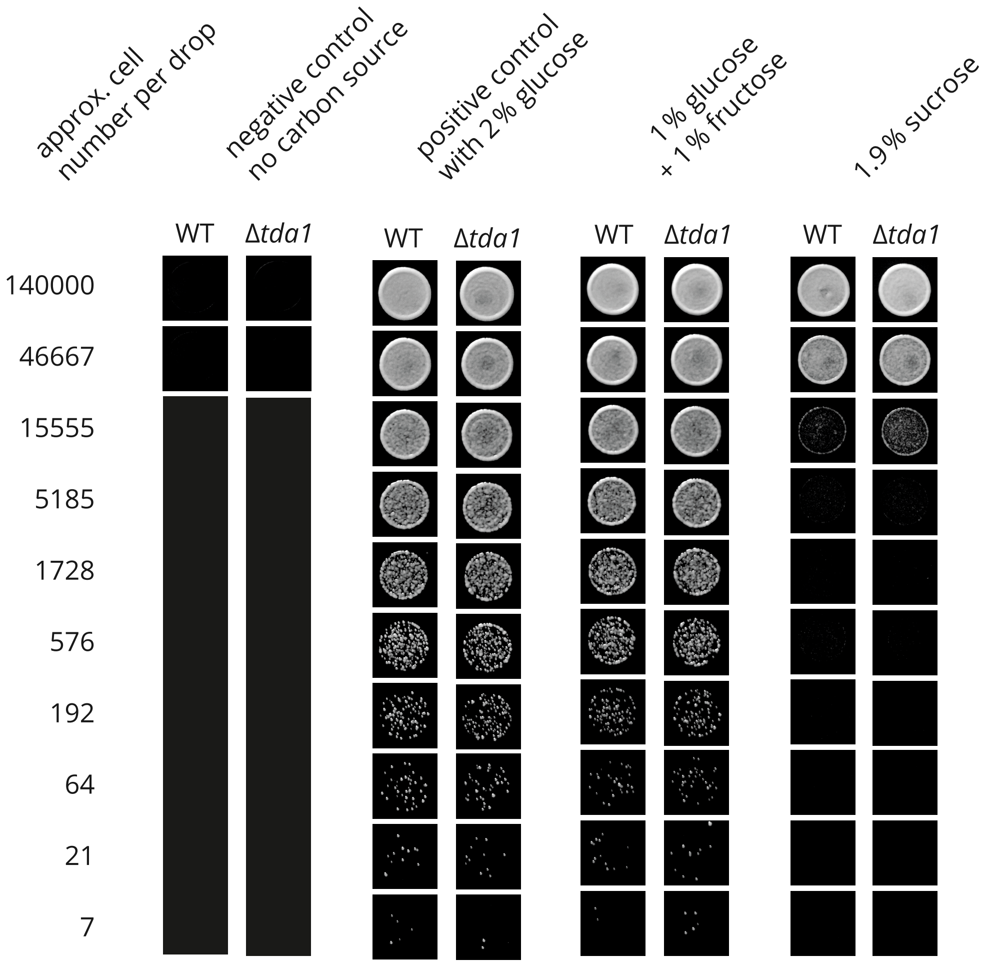

Figure S7 – Comparative assessment of the Δ*tda1* deletion mutant and the wild type growth on sucrose

Cells of the wild type and the Δ*tda1* deletion mutant were grown to their exponential growth phase in medium A containing 2 % (w/v) glucose. The cells were then washed, and 7 µl of cells in suspension were dropped on agar plates with medium A plus the indicated carbon source. The suspension was diluted so that roughly the given number of cells can be expected to be contained in one drop ($2x10^7$ cells per ml of cell suspension with an OD_{600} of 1). Equivalent amounts of fructose glucose mix and sucrose were used for the plates. Plates with 2 % (w/v) glucose were used as the positive control, plates without carbon source as the negative control (black bars where pictures and visual inspection both showed no signal). The incubation was carried out at 30 °C for 26 h. The experiment was performed three times independently. Photographs of agar plates were taken as raw image files with a DSLR camera and developed in Adobe Photoshop Lightroom Classic (making use of the functions for color correction, noise reduction and sharpening) before further processing and cropping of the individual tiles of the figure in Adobe Photoshop. Settings were chosen to optimize the visibility of all colonies present and eliminate any background.

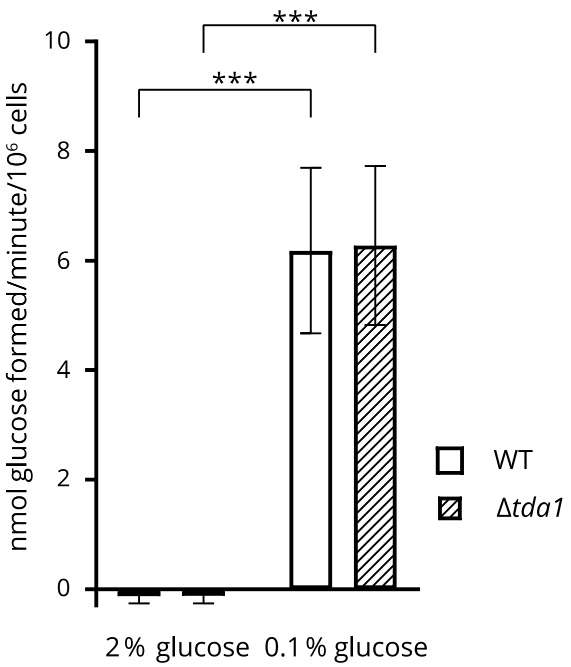

Figure S8 – Assessment of invertase activity in the Δ*tda1* deletion mutant and the wild type

The activity of secreted invertase was investigated by a colorimetric assay measuring glucose liberation from sucrose with wild type and Δ*tda1* deletion mutant cells grown for 4 h in YP medium containing 0.1 % or 2 % (w/v) glucose. The precultures had been grown at 2 % (w/v) glucose. The repression of the invertase gene can be seen at 2 % (w/v) glucose but is reduced at 0.1 % (w/v) glucose. No significant difference between the wild type and the Δ*tda1* deletion mutant was detected via ANOVA. Employing a Bonferroni multiple comparisons test, the significant influence of the glucose concentration was confirmed for the subgroups of wild type and Δ*tda1* deletion mutant post hoc (*** p ≤ 0.0001). The experiment was performed in four replicates. The error bars show the standard error of the mean.

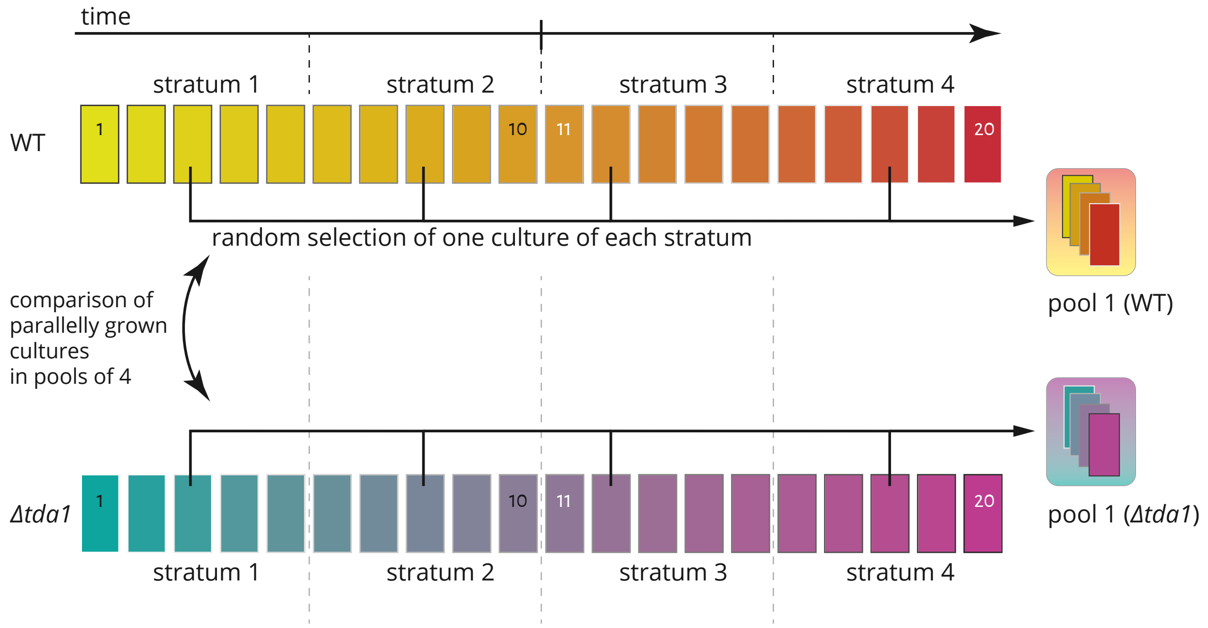

Figure S9 – Overview of the sample pooling for the 2D-DIGE experiments

The procedure that was employed for combining the protein extracts of 4 cell cultures into one pool for each condition (wild type and Δ*tda1* deletion mutant, each grown at an initial glucose concentration of 0.1 % and 2 % (w/v)) is illustrated. Only the independent variable genotype (wild type and Δ*tda1* deletion mutant) is shown, but the figure applies to the pools for 0.1 % as well as the ones for 2 % (w/v) glucose. The individual 20 cultures per condition were stratified by time, and one culture out of each of the four strata was randomly assigned to one pool. Consistently, four cultures were grown under the four different conditions in parallel. For every condition, one pool contained the same combination of randomly chosen cultures from each of the four strata, and the pairs of the wild type and Δ*tda1* deletion mutant proteomes were separated on the same gel.

Supplementary Comment

on the model-based spot quantification

In the present study, the differentiation of the two hexokinase isoforms Hxk1 and Hxk2 was successfully achieved via 2D-DIGE. Spot quantification was performed with the Melanie software and by a model-based procedure as described in the Experimental procedures section. The data from the Melanie software (supplementary Fig. S4) can be expected to be minimally biased by the investigator due to the automatic detection. However, correct quantification of overlapping spots is limited in the Melanie software and automatic determination of spot boundaries, despite very good results in general, is sometimes less than ideal. The model-based quantification, on the contrary, heavily relies on visually evaluating the obtained data, while the model does not assume any spot boundaries but relies on the assumption of symmetry to decompose molecularly heterogeneous spot entities proceeding from local minima and maxima assessed across all gels. Given that the 2D-DIGE employs two independent methods for separation according to isoelectric point and molecular size, symmetry is a rather bold assumption. However, for many spots, including the ones presented here, this assumption seems feasible and greatly simplifies the following calculations. In contrast to similar, more complex approaches described before [65], this simplification makes the method presented in the present work very accessible, particularly as the required software is available free of any charges. Relevant local maxima of potential spots were determined to define the number of Gaussian curves in the model, feasible starting points and boundaries for the parameter fitting. This does introduce some bias, as, to our knowledge, intelligent object detection in image data always does.

A big advantage of the model-based quantification method is that, as local minima and maxima are initially assessed across all gels, the true position of a spot can be determined for the condition with the least interference and then generalized to all gels. In other words, the information of all gels can be used to construct an idealized proteome spot map where each protein entity possibly identifiable by the method has a defined position on the spot map. The idea of standard positions for a protein species has initially been presented in Ref. 66. In the present study, the synthesis of the idealized proteome spot map is realized as an intuitive, manual procedure, which is visualized in Fig. 8. For future development beyond this primarily human intuition-based method of finding the idealized center position of spots, it should be relatively easy to implement an algorithm that determines the spot centers taking into account not one gel profile at a time but all of them combined in parallel (global fitting).

In the present study, the model-based quantification was specifically applied for determining the quantities of Hxk1 and Hxk2 in their serine 15 phosphorylated and unphosphorylated form, because spot detection and quantification with the Melanie software appeared suboptimal. Overall, despite some detected differences, the automated quantification with the Melanie software led to results which roughly matched the ones obtained by the likely more accurate and more reliable model-based quantification. Although this can be interpreted as validation of the model-based quantification method, it

retrospectively also suggests that the new method, despite its advantages, was not of paramount importance for the Hxk1 and Hxk2 quantification it was applied to in the present study. Nevertheless, the model-based quantification clearly offered improvements regarding the analysis of overlapping spots, and its current implementation may be a helpful and easy-to-use tool for further research conducted using 2D-gel-electrophoreses.

Applying the model-based quantification, the same model for analysis of all replicates of wild type and mutant spot patterns was used, because this approach appeared more objective than the conceivable alternative of setting a visually not distinguishable abundance of a spot to zero before analysis. Even so, a considerable problem with the taken approach is that when a spot is not present under certain circumstances, the fitting algorithm will still attempt to determine parameters for that spot which may easily lead to an overparameterized model, obviously implausible results or failure of the fitting algorithm to determine any result. To prevent these events, manual optimization of the starting and boundary values was necessary, thereby introducing a subjective judgment of which results can be considered plausible. Also, this approach increases the manual work necessary.

For quantification using the Melanie software or the model-based quantification, only the four main spots containing hexokinase were included. On the one hand, hexokinase abundance attributable to other spots in which hexokinase had been detected was low. On the other hand, one cannot exclude that even more spots might contain hexokinases, perhaps due to unknown modifications. So, unknown modifications, resulting in spots not being identified or included in the analysis, were assumed to decrease the protein amount in the considered spots equally. Furthermore, the assumption was made that the four considered spots (number 915, 916, 942 and 938) are equally affected by all errors like losses during protein extraction or electrophoresis. Although these assumptions must be considered for evaluation of the conclusions of the present study, similar assumptions must always be made for quantifications based on western blots or purely mass spectrometry-based methods as well.
